# Toward Rational Design of Aqueous Battery Membranes Beyond the Selectivity‐Conductivity Trade‐Off

**DOI:** 10.1002/advs.78245

**Published:** 2026-10-14

**Authors:** Jinkang Liu, Mingbo Xie, Zhenghao Wang, Huacun Li, Jun Xie, Pan Liu, Qingwei Wang, Xu Yan, Meiqing Shi, Liyuan Chai, Yue Luo, Zining Tang, Rui Tan, Liyuan Zhang

**Affiliations:** ^1^ School of Metallurgy and Environment Central South University Changsha Hunan P. R. China; ^2^ State Key Laboratory of Advanced Metallurgy for Non‐ferrous Metals Changsha Hunan P. R. China; ^3^ Chinese National Engineering Research Center for Control & Treatment of Heavy Metal Pollution Changsha Hunan P. R. China; ^4^ Department of Chemical Engineering Swansea University Swansea UK

**Keywords:** membrane design, proton transport ion selectivity, redox flow batteries, sulfonic‐acid‐based membranes

## Abstract

Electrochemical energy‐storage systems increasingly require membranes that can simultaneously deliver fast ion transport, suppressed active‐species crossover, chemical and dimensional stability, and scalable manufacturability. Yet conventional membrane platforms still struggle to reconcile conductivity with selectivity, while durability and processability remain important practical constraints. Sulfonic‐acid‐based membranes are particularly attractive because they combine strong acidity, fixed‐charge character, and tunable hydrated ionic domains. Together, these features make sulfonic acid groups a versatile design element for regulating transport, exclusion, swelling, and membrane processability. Although these features have been most extensively explored in redox flow batteries, the resulting insights are also highly relevant to the broader design of next‐generation energy‐storage membranes. This review therefore uses sulfonic‐acid‐based membranes in redox flow batteries as a representative platform to extract transferable design principles. It covers proton‐transport mechanisms, synthetic and microstructural design strategies, representative fluorinated and non‐fluorinated membrane classes with flexible and rigid architectures, and their roles in vanadium, zinc, and emerging organic redox flow batteries. It further discusses cost, durability, dimensional stability, scale‐up, and future opportunities in localized sulfonic‐acid placement, biomass‐derived membranes, and data‐driven design. By clarifying structure–transport–manufacturing relationships, this review extracts general design principles for advanced membranes across electrochemical energy‐storage systems.

## Graphical abstract

This review examines sulfonated membranes for redox flow batteries. It focuses on proton transport mechanism, membrane design, performance limits, optimization strategies, and the pathway from molecular design to membrane manufacturing.

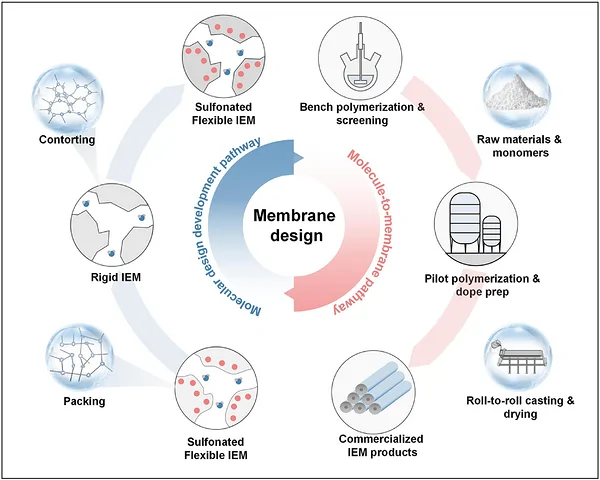

## 1 Introduction

The dual challenges of energy security and environmental sustainability are reshaping the global energy landscape. With the rapid deployment of renewable resources such as solar, wind, and hydropower, ensuring a stable and resilient electricity supply has become increasingly complex [1]. The intrinsic intermittency of these energy sources necessitates the development of large‐scale, durable, and cost‐effective energy storage technologies to enable reliable grid operation and accelerate the transition toward a low‐carbon future [2, 3]. RFBs have emerged as a promising candidate to address this need, particularly for long‐duration applications, offering scalable operation from kilowatt to megawatt levels with storage duration typically ranging from 4 to 12 h [4]. Beyond their scalability and suitability for long‐duration applications, RFBs also exhibit outstanding durability, typically sustaining over 20 000 cycles with minimal capacity loss, which makes them particularly attractive for daily cycling [5]. Their use of non‐flammable aqueous electrolytes further enhances operational safety compared with many alternative storage technologies [6]. Moreover, with current levelized costs near $0.074 kWh^−1^ and projected to fall below $0.034 kWh^−1^ by 2030 through advances in materials and manufacturing [7], RFBs are rapidly approaching cost competitiveness for grid‐scale deployment. Collectively, these attributes underscore the pivotal role of RFBs in enabling a reliable and sustainable energy transition [8].

Despite these advantages, the widespread deployment of RFBs is still constrained by the performance of key components, among which membranes are particularly critical. Serving as ion‐selective barriers, membranes enable efficient proton transport while suppressing the crossover of redox‐active species [9, 10]. However, their failure directly compromises battery efficiency and longevity [11]. The primary degradation pathways include loss of selectivity, resulting in redox species crossover [12], chemical attack under oxidative or strongly acidic conditions [13], and mechanical damage from stress or handling [14]. To overcome these challenges, ideal membranes must simultaneously meet multiple stringent criteria: low cost for economic viability, high ionic conductivity and high selectivity as the central performance target, together with strong resistance to redox‐active‐species crossover and robust mechanical and chemical stability under harsh operating environments (Figure 1a) [15]. Furthermore, using sustainable materials and fabrication processes is increasingly important [16, 17].

**FIGURE 1:**
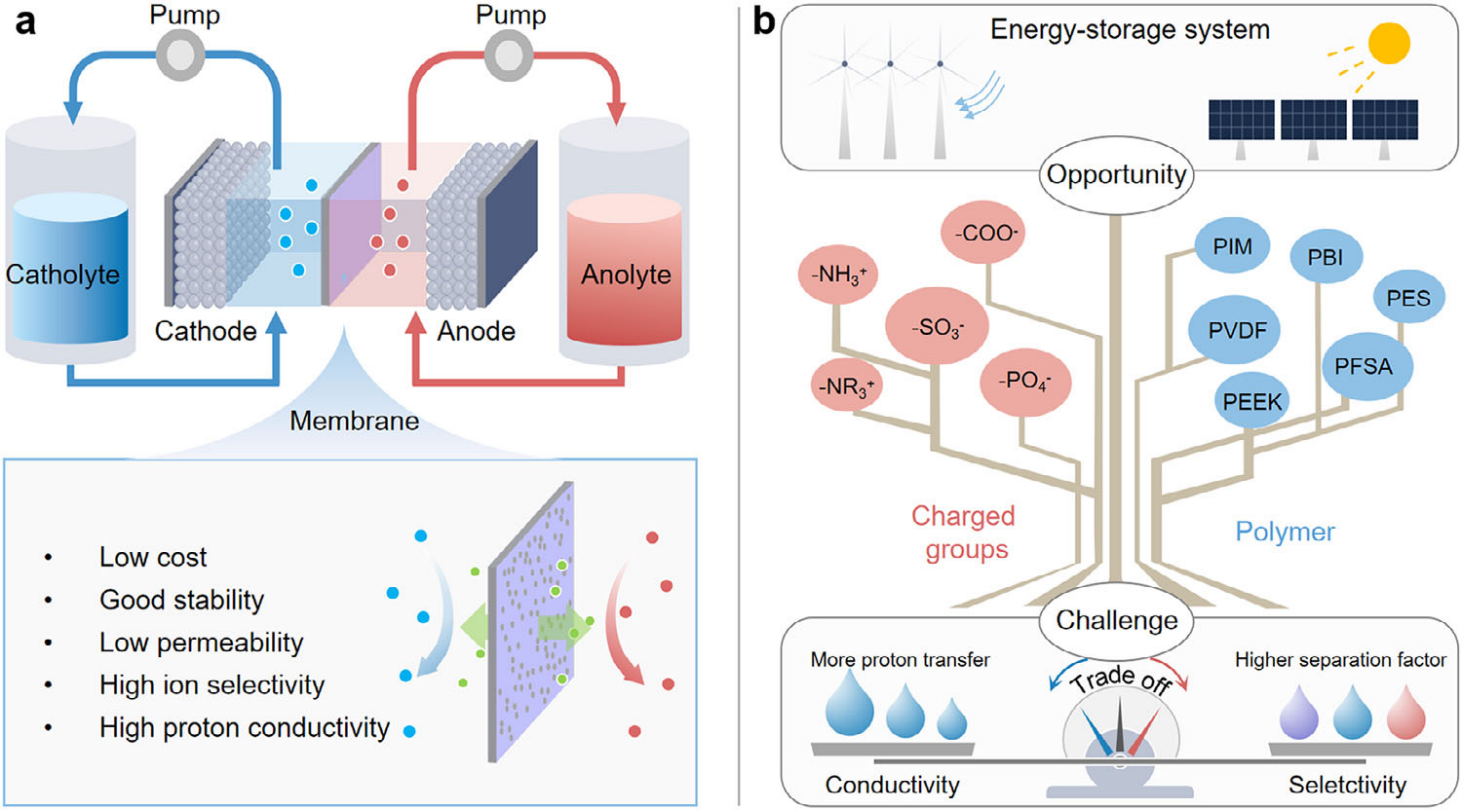
(a) Schematic of ion‐conductive membrane‐based RFBs and idealized membrane design. (b) Opportunities and challenges of membranes in RFBs.

The development of membranes for RFBs presents both significant opportunities and challenges [18]. Driven by the rapid growth of RFBs research, a wide range of polymer backbones has been investigated, including perfluorosulfonic acid (PFSA), polyimide (PI), polymer of intrinsic microporosity (PIM), polyvinylidene fluoride (PVDF), polyether ether ketone (PEEK), polyethersulfone (PES), polyether ether ketone ketone (PEEKK), polyaryletherketone (PAEK), polyarylethersulfone (PAES), and polybenzimidazole (PBI) [19]. These materials can be functionalized with various groups, such as sulfonic acid, quaternary ammonium, and zwitterionic moieties, each offering unique advantages in terms of ion selectivity, conductivity, and chemical stability (Figure 1b). Among various functional groups employed in RFBs, sulfonic acid groups (–SO_3_H) are particularly useful for forming hydrated ion‐transport pathways in acidic aqueous membranes, although their suitability depends on the battery chemistry. Compared with phosphoric acid‐containing functionalities, phosphoric acid (–PO_4_H), quaternary ammonium (–NR_3_H), and carboxyl (–COOH) groups, –SO_3_H has distinct acid dissociation and hydration characteristics. The suitability of each functionality depends on the intended charge carrier and operating electrolyte [20]. Notably, –SO_3_H groups are typically close to fully dissociated in water to release protons across a wide pH range, ensuring high ionic conductivity, while their ability to form dense hydration networks and exert strong Donnan exclusion effects substantially suppresses the crossover of multivalent cations [21]. In contrast, quaternary –NR_3_^+^ carry a permanent positive charge and can support anion transport; their chemical stability depends on the group structure, polymer backbone, and operating electrolyte [22]. Similarly, –COOH suffers from incomplete dissociation and poor stability, particularly under harsh operational conditions [23]. However, –PO_4_H are commonly used in fuel cells for proton conduction, particularly in systems that operate under high temperatures and anhydrous conditions. In these environments, phosphoric acid facilitates proton conduction through the Grotthuss mechanism, providing effective proton transport pathways at high temperatures. Additionally, the phosphoric acid‐bearing materials are of excellent thermal stability and resistance to carbonization. However, in RFBs, which often involve strong oxidative anodes or reducing cathodes, phosphoric acid‐based membranes are prone to degradation due to the attack of the phosphoester and phospho‐oxygen bonds. This leads to the leaching or migration of phosphoric acid groups, which ultimately shortens the lifespan of membrane. As a result, phosphoric acid‐based membranes are superior to sulfonic acid‐based membranes in fuel cells operating under high‐temperature, anhydrous conditions, but are not suitable for RFB membranes in strongly oxidative aqueous environments. Sulfonated polymer membranes dominate commercial applications in RFBs, whereas –NR_3_^+^ and –COOH membranes remain confined to niche scenarios with specific pH requirements [24]. The expanding library of membrane designs and functionalization strategies has opened new avenues for optimizing performance in RFBs and addressing specific operational requirements [25]. Despite these advances, a central challenge persists: achieving a balance between conductivity and selectivity [26]. High conductivity is essential for minimizing ohmic resistance, whereas high selectivity is critical for reducing crossover and preserving capacity. For most membrane systems, improving one often comes at the expense of the other [15, 27, 28]. For example, increasing membrane thickness enhances selectivity but hinders ion transport, while boosting ion‐exchange capacity (IEC) improves conductivity at the cost of greater species crossover [29]. Addressing this trade‐off requires innovative approaches such as asymmetric architectures, hybrid materials, and advanced functionalization strategies, aiming to reconcile the conductivity–selectivity conflicts without sacrificing durability [30, 31]. Continued progress along these lines will be key to unlocking the full potential of RFB membranes for large‐scale energy storage applications [32].

Building on these challenges, recent research has increasingly focused on molecular‐level design strategies to overcome the conductivity–selectivity trade‐off. This trade‐off remains the key obstacle to practical membrane performance and to the translation of membrane design from laboratory studies toward industrial applications [33, 34]. A central principle lies in tailoring the polymer backbone that can be broadly categorized into flexible and rigid architectures. Flexible backbones with rotatable linkages can be readily functionalized with ion‐conducting moieties, enabling the ionic conductivity required for practical RFB membranes [35, 36]; however, after functionalization, they often struggle to withstand solvation‐driven swelling. This vulnerability originates from their high conformational freedom, hydrated ionic groups act as strong solvating/plasticizing centers, and the mobile chains can readily reorganize to accommodate solvent uptake, resulting in pronounced dilation of free volume, excessive swelling, and a consequent loss of ion selectivity. By contrast, rigid frameworks built from contorted, shape‐persistent skeletons with restricted rotational freedom are intrinsically less susceptible to such swelling because the constrained backbone suppresses segmental rearrangement and provides a mechanically robust scaffold that limits solvent‐induced dilation, thereby maintaining dimensional stability and ion selectivity [37, 38]. Leveraging this inherent resistance, sulfonated rigid backbones have attracted particular interest because they introduce a high density of fixed acid sites while retaining the anti‐swelling characteristics of the rigid matrix, thereby offering a more favorable route to improve conductivity while not sacrificing selectivity [39]. Although –SO_3_H incorporation generally promotes hydration and ionic aggregation, the limited chain mobility and intrinsic free volume of rigid backbones can spatially confine the hydrated ionic domains and stabilize the percolated ionic network, mitigating over‐swelling while sustaining selective ion transport. Meanwhile, interconnected ionic clusters provide continuous proton‐transport pathways, leading to improved overall performance when the degree of sulfonation (DS) and the microphase‐separated morphology are judiciously regulated. Consequently, such sulfonated rigid membranes can shift membrane performance beyond the conventional conductivity–selectivity limitation. They also support high‐current‐density operation and superior long‐term cycling stability relative to conventional fluorinated systems [40]. Furthermore, translating these molecular‐level design principles into practical membranes requires a well‐orchestrated molecular design‐to‐membrane fabrication and manufacturing pathway. Beginning with rational molecular design and polymer synthesis, the process advances through controlled membrane casting, crosslinking, and structural optimization to achieve the desired morphology and performance [41, 42]. Subsequent scale‐up involves adapting laboratory fabrication methods, such as solution casting or phase inversion, to continuous roll‐to‐roll production, while ensuring uniform thickness, mechanical robustness, and consistent ion transport characteristics [43]. This integrated pathway bridges fundamental molecular design with industrial implementation, paving the way for the large‐scale deployment of high‐performance sulfonated membranes in energy conversion and storage technologies (Figure 2).

**FIGURE 2:**
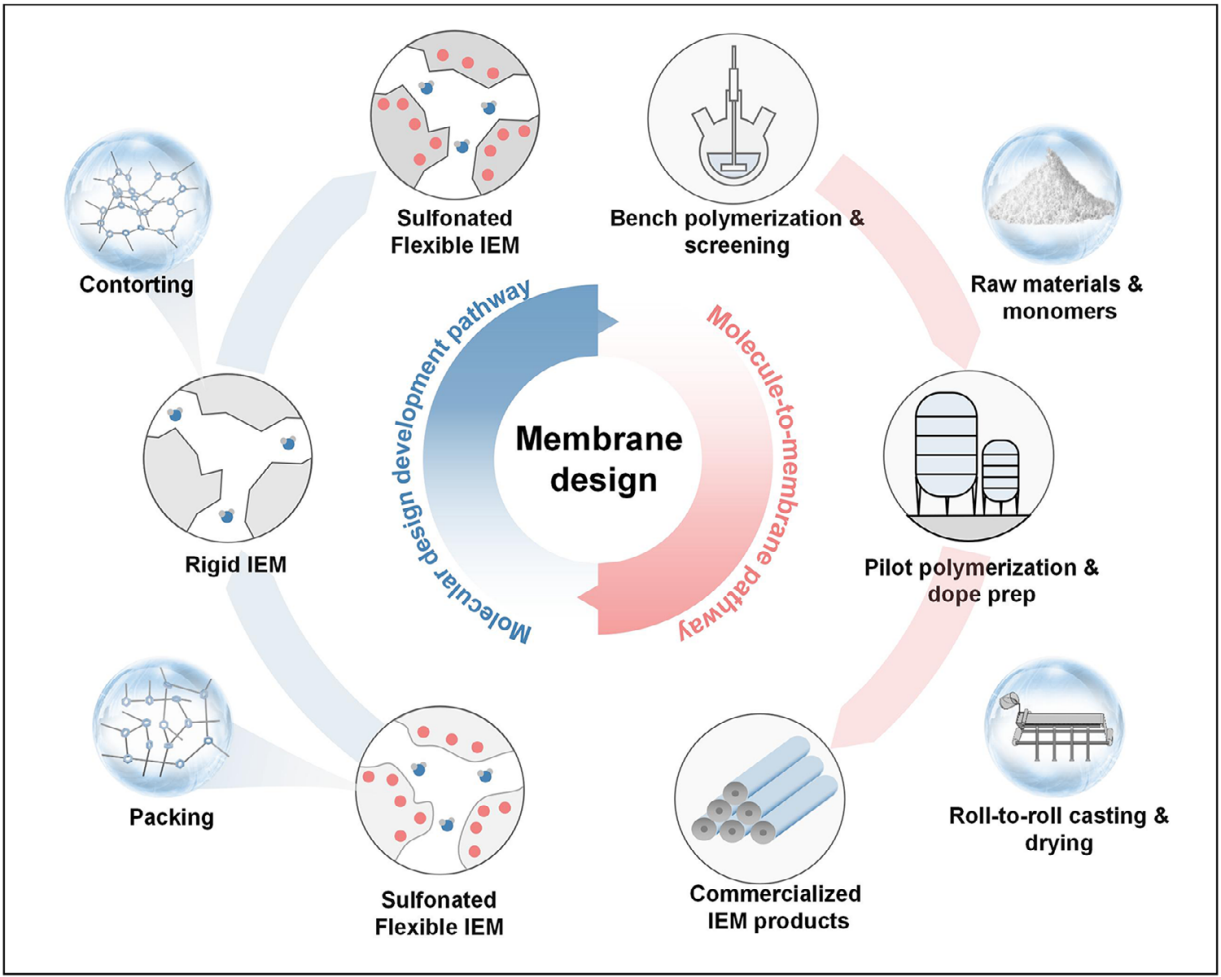
Schematic of the development of a versatile membrane family for RFBs, including the development pathways of sulfonated flexible/rigid membranes and membrane commercialization.

The development of RFBs membranes has been shaped by several pivotal milestones. The initial concept was established in 1950 with the synthesis of early ion‐exchange membranes [44], followed by the demonstration of a Fe–Cr RFBs prototype in 1974 [45], which defined the basic system architecture. A significant breakthrough came in 1978 with the advent of Nafion, whose microphase‐separated channels set the benchmark for proton conduction [46]. These advances have since catalyzed the exploration of sulfonic acid‐functionalized membranes, which now represent the state of the art in a variety of RFBs [47]. Figure 3 highlights the representative achievements of sulfonated polymeric membranes across vanadium, zinc, and organic RFBs systems. Despite this progress, systematic analyses remain limited. Existing reviews [24, 48, 49] have typically summarized the types and applications of sulfonated polymeric membranes, but have less often examined how –SO_3_H directly addresses the conductivity–selectivity trade‐off while simultaneously influencing durability and manufacturability. Unlike a classification based solely on polymer families or battery systems, an –SO_3_H‐centered perspective provides a common chemical variable for comparing different membrane platforms. The concentration and spatial distribution of –SO_3_H, together with its interactions with the surrounding matrix, influence hydration, ionic‐domain organization, ion transport, and swelling.

**FIGURE 3:**
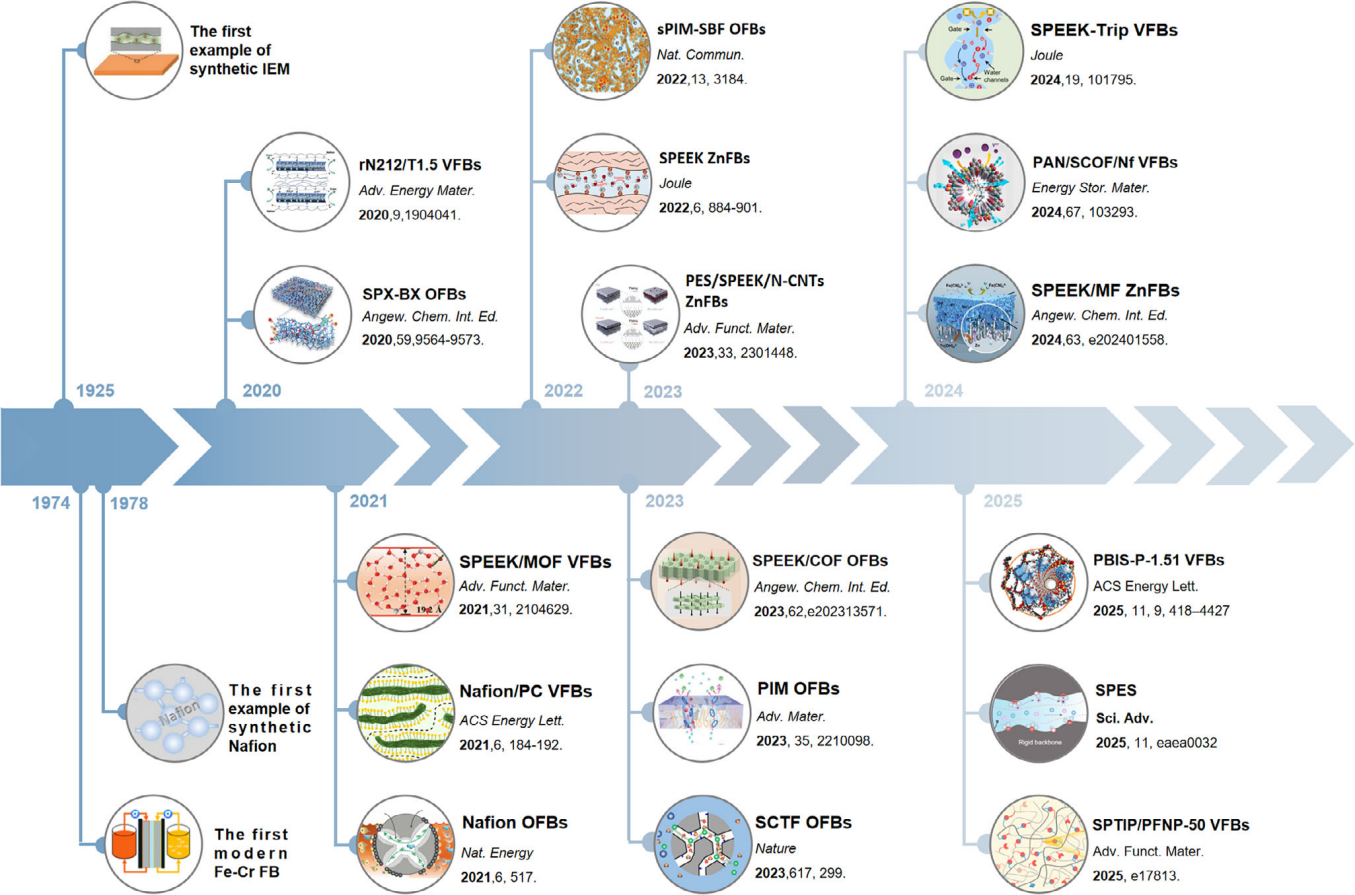
Timeline of RFB milestones and sulfonated polymer advances in components.

This review provides a comprehensive analysis of sulfonated polymeric membranes for RFBs, beginning with an overview of membrane requirements, key characteristics, and recent research progress. Here, –SO_3_H serves as the organizing chemical motif, whereas backbone composition and flexibility provide the structural context for comparing its effects across membrane families. Particular attention is given to the pivotal role of sulfonic acid groups in regulating ion transport. The conductivity–selectivity trade‐off is treated as the central design challenge, and the structure–property relationships underlying this trade‐off are systematically examined. The review further compares representative membrane families and summarizes their applications in vanadium redox flow batteries (VRFBs), zinc redox flow batteries (ZnRFBs), and organic redox flow batteries (ORFBs). Here, ORFBs refers to systems containing organic redox‐active species; these may use aqueous electrolytes and should not be conflated with non‐aqueous RFBs. The latter are considered briefly to define the applicability limits of the aqueous membrane design principles discussed in this review. On this basis, we offer an in‐depth perspective on future research directions to address these limitations and accelerate the translation of sulfonated polymeric membranes from laboratory studies to large‐scale deployment.

## 2 Advantages of –SO_3_H and Its Role in RFBs Membranes

In this section, –SO_3_H is discussed not simply as a proton‐conducting group, but as the central functional motif through which conductivity, selectivity, and structural stability can be co‐regulated. Sulfonated polymers can be prepared by either post‐sulfonation of a preformed polymer backbone or polymerization of pre‐sulfonated monomers, thereby introducing –SO_3_H groups as fixed ionic functionalities within the macromolecular framework. The hydrophilic nature of –SO_3_H effectively enhances proton conduction [50]. Beyond hydrophilicity, –SO_3_H enhances proton conduction by virtue of its strong acidity and fixed –SO_3_^−^ charge. The near‐complete dissociation supplies mobile protons and stabilizes hydrated ionic domains, while the sulfonate‐proton pair interaction remains loosely associated enough to avoid excessive proton trapping and preserve high mobility. By contrast, membranes containing strong proton‐accepting groups that promote tight acid–base association tend to immobilize protons as tight ion pairs, which reduces the population of mobile carriers and increases the activation energy for proton transport, ultimately lowering conductivity especially under low hydration. In this sense, the importance of –SO_3_H lies not simply in raising conductivity, but in providing a functional basis for improving proton transport without inherently sacrificing selectivity.

Figure 4a summarizes the correlation between ionic conductivity and permeability for polymer‐based proton‐exchange membranes reported in recent years [9, 51, 52, 53, 54, 55, 56, 57, 58, 59, 60, 61, 62, 63, 64, 65, 66, 67, 68, 69, 70, 71, 72, 73, 74, 75, 76, 77, 78, 79, 80], thereby visualizing the conductivity–selectivity trade‐off that defines membrane performance in RFBs. Notably, the permeability data are recorded using two representative active species, namely vanadium ions and ferricyanide/ferrocyanide couples, enabling a side‐by‐side comparison of transmembrane transport characteristics across different chemistries. On this basis, the figure compiles a set of representative includes representative membrane systems and introduces an empirical upper bound as a benchmark, which reflects the best‐achievable conductivity at a given permeability level. Overall, the dataset indicates that –SO_3_H‐functionalized membranes tend to cluster closer to, or even surpass, this empirical upper bound, indicating that sulfonation is one of the key factors that can improve conductivity without proportionally increasing crossover. For instance, representative systems such as SPIM [81] and SPEEK‐Trip [82], illustrate this trend clearly. In both cases, increasing sulfonation density promotes the formation of hydrophilic ionic domains and continuous proton‐transport pathways, thereby enhancing conductivity without a proportional loss in crossover resistance. As a result, the data shift toward the upper‐bound region, showing that properly regulated sulfonation can move membrane performance beyond the conventional conductivity–permeability trade‐off. A similar tendency is now emerging in ferricyanide‐based systems. Notably, in sulfonated cation‐exchange membranes, the fixed –SO_3_^−^ groups exert strong electrostatic exclusion against the anionic ferricyanide/ferrocyanide redox couple, thereby suppressing active‐species permeation while maintaining high ionic conductivity. This comparison suggests that the role of sulfonation is not limited to vanadium systems, but may provide a more general route to improving the transport–selectivity balance across different redox chemistries.

**FIGURE 4:**
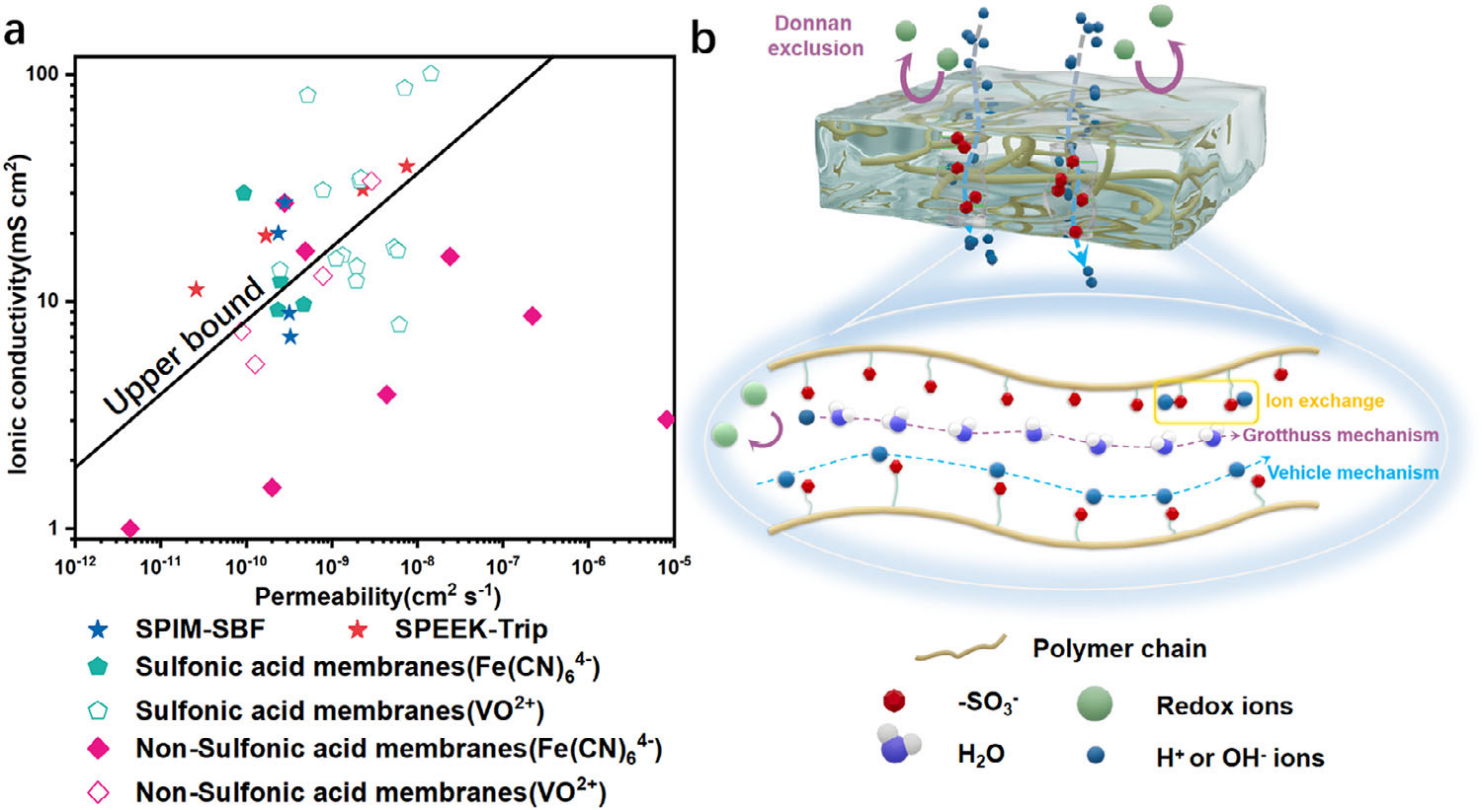
(a) A plot of ionic conductivity versus permeability for representative membranes. (b) Schematic demonstration of ion transport mechanisms through sulfonated‐based membranes.

Proton transport in sulfonated membranes proceeds through three synergistic pathways: (i) ion exchange from dissociated –SO_3_H groups, which increases the pool of mobile protons and concomitantly raises IEC; (ii) vehicle transport, whereby hydrated proton complexes diffuse through water‐rich ionic domains; and (iii) Grotthuss hopping, in which protons migrate along extended hydrogen‐bond networks (Figure 4b). Increasing the –SO_3_H content amplifies these conduction modes by enhancing hydration and promoting the development of percolated ionic networks, thereby elevating conductivity. At the same time, –SO_3_H groups can strengthen electrostatic exclusion and foster a more discriminating ionic‐channel architecture, which suppresses active‐species permeation and improves selectivity, provided that swelling is effectively regulated. In this regard, rigid backbones offer a critical structural advantage. Their shape‐persistent frameworks confine hydrated ionic domains into well‐defined, continuous transport channels and suppress solvent‐uptake‐driven swelling, thereby retaining the connectivity required for efficient proton conduction without undermining ion selectivity. Consequently, judiciously engineered sulfonation, particularly within rigid matrices, can shift membrane performance toward breaking the conductivity–permeability trade‐off.

The Nernst–Planck equation provides a theoretical framework to quantitatively describe the synergistic enhancement of ion transport by –SO_3_H [83]. As shown in Equation (1):

(1) $$\bar{J_{i}}=-D_{i}\Delta c_{i}-\frac{z_{i}F}{\mathrm{RT}}D_{i}c_{i}\Delta \phi$$

where $\bar{J_{i}}$, ***D***_***i***_, ***c***_***i***_, ***z***_***i***_, and ϕ are the ion flux, diffusion coefficient, ion concentration, valence of ionic species, and chemical potential, respectively. F, R and T are the Faraday constant, universal gas constant, and Kelvin temperature, respectively.

According to Equation (1), the –SO_3_H enhances ion transport through multiple, interrelated mechanisms. First, their strong hydrophilicity promotes the formation of continuous nanochannels [84], which markedly increase the diffusion coefficient (***D***_***i***_) of charge carriers [85]. Simultaneously, the fixed negative charges of –SO_3_H intensify the local chemical potential gradient (ϕ) within the migration term, thereby increasing the electromigration driving force and enhancing the migration contribution to the overall ionic flux. In addition, the Grotthuss mechanism provides an extra contribution to proton conduction, enabling proton hopping between adjacent –SO_3_H sites through hydrogen‐bonded water networks, potentially assisted by quantum tunneling. Collectively, these effects couple nanochannel formation, electrostatically driven migration, and proton hopping, while the fixed –SO_3_^−^ charges generate a Donnan potential at the membrane interface that shifts ion partitioning and lowers the membrane‐phase concentration of co‐ions, thereby reducing co‐ion transport and improving selectivity.

Figure 4 links the conductivity–permeability balance to the transport role of –SO_3_H/–SO_3_^−^ in hydrated membranes. This role should be compared with the requirements of the electrolyte rather than used to rank functional groups universally. Fixed –SO_3_ sites can support cation transport, whereas fixed –NR_3_^+^ sites can support anion transport. The contribution of –COOH depends on its ionization under the operating conditions, while phosphoric acid‐containing and zwitterionic groups provide different local environments for ion association and hydration. Selecting among these functionalities therefore requires identifying the intended charge carrier, the active species to be retained, and the stability of the complete membrane in the operating electrolyte.

Because –SO_3_H can simultaneously regulate transport, exclusion, and swelling, its incorporation into diverse material matrices has generated a broad spectrum of membrane platforms, ranging from conventional polymers to advanced composites and nanocomposites [86]. Classical and widely studied systems, including sulfonated poly(arylene ether ketone) (SPAEK), sulfonated poly(phthalazinone ether sulfone) (SPPES), sulfonated polyimide (SPI), sulfonated poly(phenylene oxide) (SPPO), PFSA, and sulfonated poly(ether sulfone) (SPES) (Figure 5), typically exhibit high ionic conductivity and robust chemical stability, arising from the synergistic interplay between –SO_3_H moieties and engineered polymer backbones [87]. As shown on the left side of Figure 5 (from top to bottom), these representative backbones are arranged in the order of SPAEK, SPPES, SPI, SPPO, PFSA, and SPES. Beyond these benchmarks, a growing set of emerging sulfonated architectures, including sulfonated poly(thioether ketone) (SPTK), sulfonated triazine framework (SCTF), sulfonated polybenzimidazole (SPBI), sulfonated polymers of intrinsic microporosity (SPIM), sulfonated poly(ether ether ketone) (SPEEK), and sulfonated poly(phenylene sulfide sulfone) (SPSS), has further expanded the design landscape, enabling more precise regulation of ion‐transport pathways and, in many cases, selectivity. On the right side of Figure 5 (from top to bottom), these emerging architectures are arranged in the order of SPTK, SCTF, SPBI, SPIM, SPEEK, and SPSS. Notably, the conformational freedom of aromatic backbones, particularly the rotatability of phenyl rings, can strongly influence chain packing, free‐volume evolution, and the continuity of ionic domains. In this regard, rigid polymer architectures generally rely on conformationally constrained and non‐rotatable phenyl units to suppress segmental rotation and maintain backbone rigidity. Accordingly, in Figure 5, non‐rotatable phenyl rings are highlighted in blue as a hallmark of rigid frameworks, whereas rotatable phenyl rings are highlighted in red to indicate more flexible linkages. In aggregate, these designs reflect a unifying rationale, leveraging –SO_3_H functionalization in concert with backbone rigidity/flexibility modulation and hybrid‐material incorporation to concurrently enhance ionic conductivity, maintain mechanical robustness, and improve ion selectivity. Figure 5 summarizes representative chemical structures of sulfonated building blocks, which are discussed below from four interrelated perspectives: chemical functionalization, structural design, composite engineering, and processing methodologies. These molecular structures provide the chemical basis for membrane design, but their transport properties ultimately depend on how sulfonation and fabrication convert them into electrolyte‐equilibrated ionic domains. The following section therefore examines the processing variables that determine whether the intended molecular design is retained in the final membrane [88].

**FIGURE 5:**
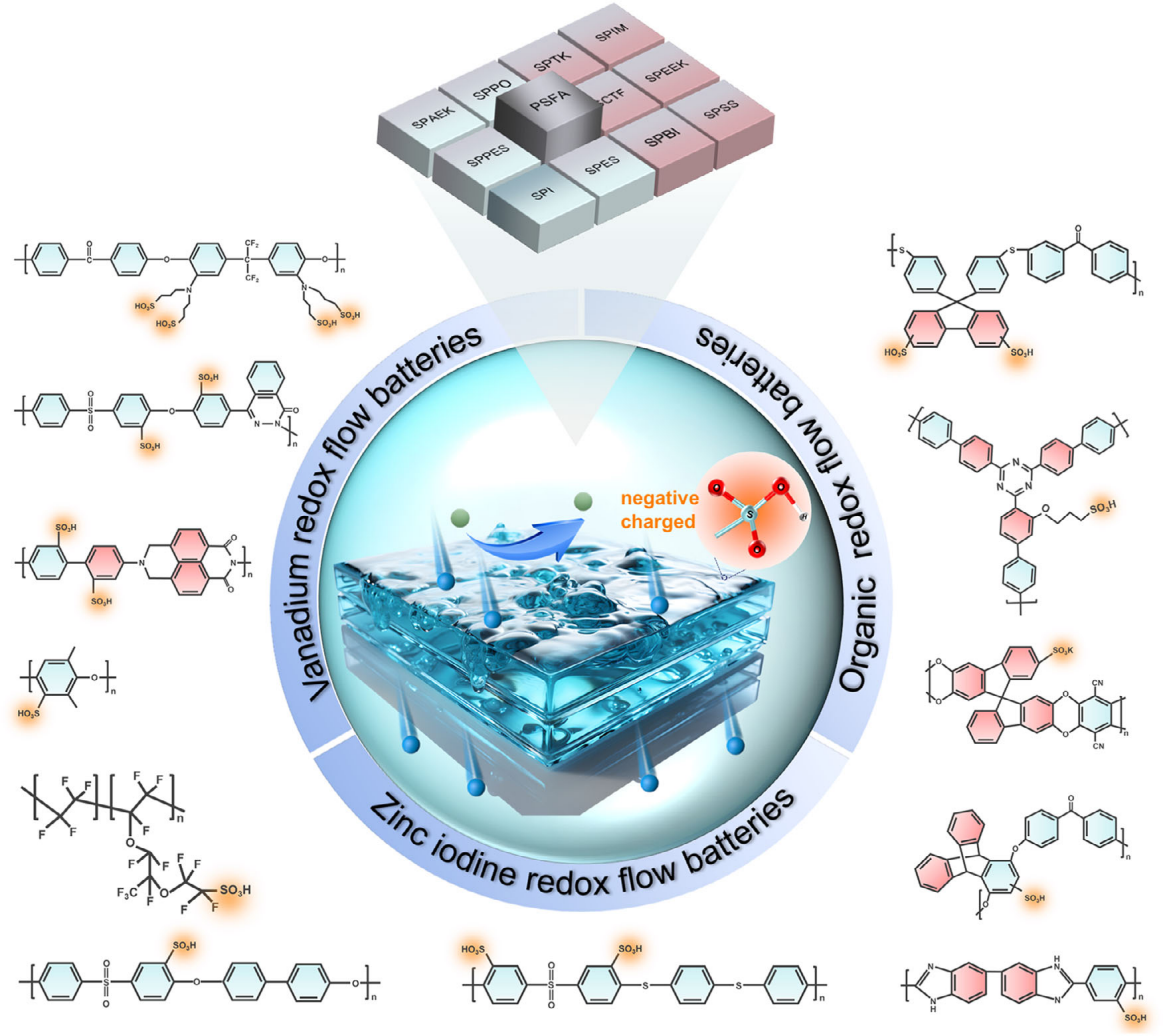
Schematic of chemical structures of sulfonated polymer for RFB membranes.

The transport principles discussed above require qualification in non‐aqueous electrolytes. Following ion exchange, sulfonate sites may conduct supporting‐electrolyte counterions rather than protons; the extent of transport depends on solvent uptake, ion solvation and association, and interactions with the polymer matrix. Water‐mediated proton conduction in acidic aqueous membranes therefore cannot be assumed to describe transport in aprotic solvents. Suitability also depends on the stability of the complete membrane–electrolyte combination, including solvent‐induced swelling or dissolution, retention of the polymer backbone and sulfonate linkage, and compatibility with the redox species and operating potential range. Conductivity and stability measured in water alone cannot establish performance in a non‐aqueous cell.

## 3 Challenges in Sulfonation Processes and Membranes Fabrication

While diverse sulfonation strategies and membrane fabrication techniques provide a versatile toolbox for tuning the structure and chemistry of membranes, realizing high‐performance systems for aqueous batteries requires a more fundamental understanding of the material and processing variables that dictate membrane behavior under realistic operating conditions [89]. These include the –SO_3_H content and its spatial distribution, the resulting water‐uptake and swelling propensity, and the nano‐to‐microstructural features of ionic domains that jointly govern conductivity and crossover [90]. Accordingly, the optimal sulfonation route should be selected based on the targeted balance among conductivity and selectivity, with dimensional stability and manufacturing feasibility treated as essential practical constraints, rather than DS alone [91]. This insight motivates a closer examination of the major sulfonation strategies, with emphasis on how each route balances synthetic practicality against control over –SO_3_H placement and the resulting membrane performance.

The development of sulfonated aromatic polymers for membrane‐based ion separation in aqueous batteries, where ion exchange membranes (IEMs) regulate charge carrier transport while limiting active species crossover, has been enabled by a range of sulfonation strategies, each offering distinct benefits and inherent limitations [92]. Broadly, the choice of sulfonation route primarily determines how –SO_3_H groups are incorporated and distributed, thereby shaping ionic‐domain morphology and ultimately controlling conductivity, selectivity, dimensional stability, and durability [93] Direct sulfonation, typically employing strong acids such as sulfuric acid or chlorosulfonic acid, is the most widely adopted method due to its operational simplicity and cost‐effectiveness, particularly for high‐performance engineering polymers such as PEEK. However, precise control over the DS is often difficult to achieve, and excessive sulfonation may induce backbone degradation or undesirable side reactions, such as crosslinking [94]. To control the sulfonation degree, indirect synthetic routes have been widely explored. Grafting sulfonated moieties onto the polymer backbones enables more precise spatial control over –SO_3_H distribution, yet it typically involves multistep reactions that increase time and cost and restrict large‐scale implementation [95]. Likewise, polymerization of pre‐sulfonated monomers offers well‐defined architectures with tailored DS and tunable microphase‐separated morphologies, but would be limited by high monomer synthesis costs and steric hindrance that compromises polymerization efficiency. Beyond polymer backbones, sulfonating inorganic building blocks has also emerged as a viable route to introduce –SO_3_H into organic–inorganic composite membrane systems. In this context, the sulfonated inorganic phases are primarily incorporated as functional fillers within a polymer matrix, supplying additional acid sites and creating interfacial ion‐transport pathways [96]. Meanwhile, the inorganic scaffold can restrain polymer‐chain relaxation and limit solvent uptake, thereby improving dimensional stability by suppressing excessive swelling. Taken as a whole, these strategies offer complementary pathways for tailoring membrane structure and performance; nevertheless, reconciling the trade‐offs among conductivity, selectivity, chemical stability, and manufacturing cost remains a central challenge for practical deployment [15, 97].

Fabrication techniques exert a profound influence on the structural and functional properties of IEMs, thereby shaping their overall electrochemical performance [98]. Solution casting, the most widely adopted approach, typically starts from preparing a homogeneous polymer via dissolving sulfonated aromatic polymers in suitable polar aprotic solvents, followed by filtration and degassing, controlled casting, and solvent removal to form dense films. Importantly, membrane structure is not determined by dissolution alone; rather, the evaporation parameters, including temperature, humidity, and drying rate, together with solution concentration and viscosity and subsequent post‐treatments such as thermal annealing, acid exchange, and hydration equilibration, collectively govern phase‐separation kinetics, ionic‐domain connectivity, defect formation, and dimensional stability. Consequently, careful control of these processing variables is essential for tuning the conductivity–selectivity balance and ensuring reproducible, device‐relevant membrane performance [99]. While simple and scalable, this approach often suffers from residual solvent retention, which can compromise mechanical integrity. Electrospinning, in contrast, generates nanofibrous membranes with high porosity and interconnected proton transport pathways, offering superior conductivity [100]; however, post‐treatment steps such as crosslinking are typically required to reinforce weak inter‐fiber junctions. Phase separation, utilizing solvent‐nonsolvent systems, enables the construction of tunable porous architectures that are particularly advantageous for applications like fuel cells [101]. Yet, pore collapse during solvent evaporation or drying demands careful optimization of processing conditions. Interfacial polymerization offers an alternative pathway, enabling the fabrication of ultrathin composite membranes with highly selective surface layers, such as sulfonated polysulfone–polyamide hybrids. This technique, however, requires precise control of interfacial reaction kinetics to prevent defects and ensure reproducibility. Taken as a whole, each fabrication method emphasizes distinct priorities, e.g., scalability in solution casting, architectural control in electrospinning, pore engineering in phase separation, and composite functionality in interfacial polymerization. The choice of fabrication route must therefore be carefully aligned with the targeted application, as the interplay between method and material ultimately dictates membrane performance in energy conversion and storage (Figure 6). Because processing can also alter hydration, morphology, and defect formation, Section 4 considers how these features and their transport consequences can be characterized.

**FIGURE 6:**
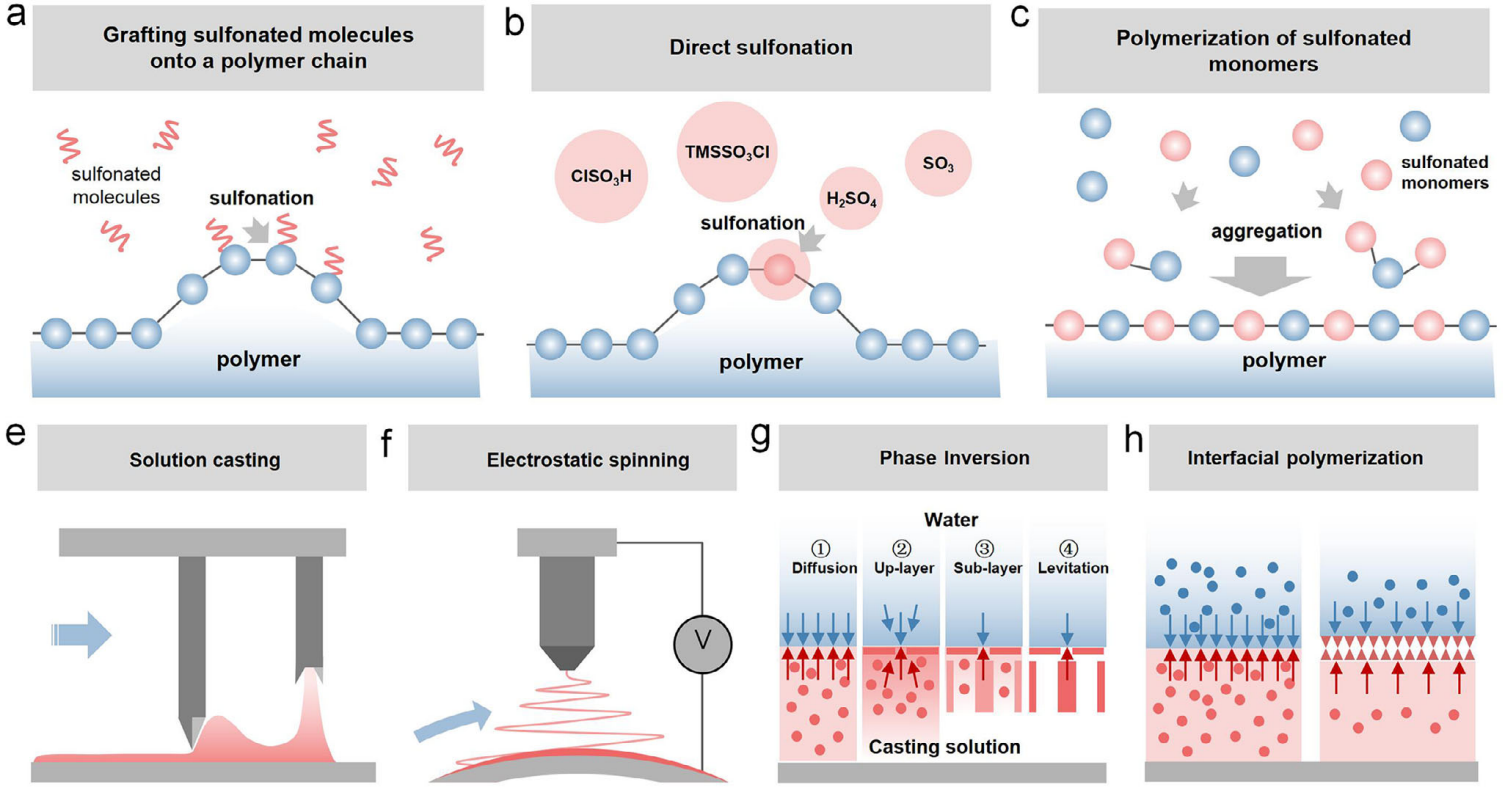
Schematic of common sulfonation methods and membrane fabrication techniques, the sulfonation methods, listed sequentially from left to right, are as follows: (a) direct sulfonation, (b) grafting of sulfonated molecules onto a polymer chain, and (c) polymerization of sulfonated monomers; The membrane preparation, from left to right, are: (d) solution casting, (e) electrospinning, (f) interfacial polymerization, (g) phase separation, and (h) interfacial polymerization.

## 4 Characterization of High‐Performance Membranes and Associated Challenges

Optimization of the membrane performance under operating conditions is a prerequisite step toward practical deployment of RFBs. This optimization requires careful control of several interrelated parameters, including IEC, water uptake, dimensional swelling, ionic conductivity, ion selectivity, mechanical robustness, and long‐term chemical/ electrochemical stability. A comprehensive understanding of these factors requires a rational design framework, which correlates composition, sulfonation level and distribution, and morphology with IEC, swelling, transport, and durability, and also relies on advanced characterization techniques capable of accurately probing structure–property relationships. This section first details these fundamental performance metrics and the standard methods used to evaluate them. We then discuss the persistent challenge of improving membrane performance beyond the conventional conductivity–selectivity trade‐off while retaining durability and manufacturability [102].

### 4.1 Conductivity

During the charge‐discharge cycling of RFBs, the energy barriers arising at the electrolyte/membrane interface and within the membrane bulk phase exacerbate ohmic polarization and energy dissipation [103]. Accordingly, achieving high power density and voltage efficiency requires minimizing internal resistance, which is fundamentally governed by membrane proton conduction [104]. Two‐electrode electrochemical impedance spectroscopy (EIS) is commonly used to determine membrane resistance and, with appropriate geometry and background corrections, ionic conductivity (σ), typically through a standardized protocol in which the membrane is sandwiched in a symmetric coin‐cell configuration and subjected to EIS measurements, followed by equivalent‐circuit fitting to extract the area‐specific resistance. Conductivity is usually calculated as:

(2) $$\sigma \;=\frac{L}{A\left(R_{1}-R_{2}\right)}\;$$

where L is the thickness of the membranes, A is the effective area of the membranes, and R_1_ and R_2_ are the resistances with and without the membranes, respectively. In addition to overall conductivity, it is important to distinguish between through‐plane ionic conductivity and in‐plane conductivity, as they describe different directions of ion transport.

Through‐plane ionic conductivity describes ion transport across the membrane thickness in RFBs. As a result, it directly maps onto the membrane's areal resistance and is the most relevant conductivity metric for estimating device‐level ohmic losses, power density, and voltage efficiency. In contrast, in‐plane ionic conductivity quantifies ion transport parallel to the membrane surface and is typically not the dominant pathway in conventional RFBs operation. Nevertheless, in‐plane measurements remain informative for materials development and diagnostics, particularly for anisotropic membranes, composite configurations, or interfacial modification layers, where lateral continuity of ionic domains near the surface can influence local transport bottlenecks. Accordingly, reporting both through‐plane and in‐plane conductivities can provide a more complete picture of transport anisotropy and morphology–property links, while through‐plane conductivity should be prioritized when correlating membrane properties with full‐cell performance. Reported conductivities should be compared only after considering membrane pretreatment, electrolyte composition, temperature, hydrated thickness, and measurement direction. Differences in these conditions may produce apparently conflicting results even when the underlying membrane behavior is similar.

### 4.2 Mechanical Properties

The mechanical integrity of IEMs is quantitatively evaluated through two key mechanical indicators: tensile strength and Young's modulus. During stack assembly and operation, membranes are subjected to multiaxial mechanical stresses originating from stack assembly pressures and flow channel constraints, necessitating superior tensile strength to mitigate fracture risks and maintain interfacial integrity. Compromised mechanical performance often stems from intrinsic material deficiencies in solution‐cast membranes, particularly when phase separation kinetics are poorly controlled during film formation or when excessive nanofillers are added but remain poorly compatible with the polymer matrix and weakly bonded at the interface. So they fail to reinforce the membrane and instead act as stress concentrators, which is a common challenge in Nafion‐based composite membranes [105].

Under practical operating conditions, especially in hybrid RFB configurations, membranes must also withstand cyclic pressure fluctuations and electrochemical swelling forces. This requirement becomes particularly stringent in metal‐based systems such as ZnRFBs, where insufficient membrane toughness increases the probability of dendrite penetration through pinhole defects, potentially leading to catastrophic short‐circuit failure. The characterization of the mechanical properties is mainly carried out using a tensile testing machine. The tensile strength can be calculated using Equation (3), as follows:

(3) $$T\;=\frac{F_{m}}{W\cdot L}\;$$

where T refers to the tensile strength of the membranes, **F**_**m**_ is the maximum tensile stress, W is the width of the sample, and L is the thickness of the membranes. The same stress–strain curve also yields Young's modulus as the slope in the initial elastic regime, providing a bulk‐averaged stiffness that is directly relevant to macroscopic deformation and sealing integrity.

While macroscopic tensile tests report bulk averaged strength and modulus, membrane microstructures are often mechanically heterogeneous due to phase separated ionic domains and filler matrix interfaces, and this heterogeneity can be overlooked by bulk testing alone [106]. Atomic force microscopy (AFM), which couples nanoscale imaging with force distance spectroscopy and can be operated in air or liquid, has therefore been used to map surface topography together with local mechanical contrast. From force distance curves, AFM force mapping can extract stiffness related metrics using contact mechanics analysis, enabling softer ion rich regions, stiffer polymer rich matrix domains, and weak interfacial zones around poorly compatibilized fillers or densified skin layers to be distinguished [107]. At the same time, direct AFM based mechanical quantification remains relatively uncommon for flow battery membranes, in part because the apparent modulus is strongly method dependent. Accordingly, AFM derived moduli should not be treated as equivalent to tensile moduli unless the contact model and deformation regime are carefully justified. Representative studies outside the RFB field illustrate both the capability and the caveats. For example, Muslimova et al. [108] utilized AFM to test the elastic modulus of a synthetic membrane used for water−oil separation. Zheng et al. [109] in situ evaluated mechanical strength of their prepared MGMs in water through nanoindentation by AFM. Overall, AFM mapping is best viewed as a complementary tool that localizes mechanical weak points relevant to cracking and sealing, and it is most informative when interpreted alongside bulk mechanical tests and other probes in a consistent assessment framework.

### 4.3 IEC

During electrochemical cycling, efficient transport of charge carriers across the membrane is essential to minimize ohmic losses and achieve high voltage efficiency (>90%) and optimize battery performance. However, excessive ion exchange capacity (IEC, typically >2.5 meq g^−^^1^) can lead to undesirable crossover of active species (e.g., vanadium ions), resulting in rapid capacity decay (>0.5% per cycle) and Coulombic efficiency loss. Therefore, precise modulation of IEC values is critical to strike an optimal balance between high ionic conductivity and acceptable crossover rates [110].

The IEC of polymer membranes is quantitatively determined through a standardized back‐titration protocol: (i) Dry membranes samples are equilibrated in 1 m NaCl solution for 24 h to ensure complete proton‐sodium ion exchange; (ii) The released protons are then titrated against 0.01 m NaOH using phenolphthalein as a pH indicator; (iii) The IEC is calculated via Equation (4):

(4) $$\mathrm{IEC}\;=\frac{C_{\mathrm{NaOH}}\cdot V_{\mathrm{NaOH}}}{W_{d}}\;$$

where C_NaOH_ is the concentration of NaOH solution and V_NaOH_ is the volume of NaOH solution consumed in titration. W_d_ is the weight of the protonated dry membranes.

### 4.4 Permeability

Membrane permeability refers to the transport rate of active species (e.g., metal ions, redox‐active molecules) across the separator under a concentration gradient, typically expressed as the area‐normalized flux of ions or molecules [111]. Excessive permeability can lead to crossover of active species, thereby reducing the Coulombic efficiency and cycling stability of the battery. Therefore, accurate quantification of membrane permeability is crucial for the design and optimization of separators in RFBs. Currently, permeability is primarily measured using a diffusion cell method, where the concentration change of active species in the receiving chamber is determined via UV–vis spectroscopy or inductively coupled plasma (ICP) techniques in an H‐type diffusion cell. Generally, the permeability of the membranes can be calculated by the equation below.

(5) $$V\;\frac{dC_{t}}{\mathrm{dt}}=\frac{A\cdot P}{L}\;\left(C_{0}-C_{t}\right)$$

where P represents the permeability of ions; L is the thickness of the test membranes; V refers to the volume of the solution; A is the effective area of the membranes; and ***C***_0_ and ***C***_***t***_ are the initial concentration of the active species, the concentration of sampling solution at time t, respectively.

It should be noted that, although the H‐type diffusion‐cell method has been widely adopted to quantify membrane permeability, it essentially probes zero‐field, concentration‐gradient‐driven transmembrane diffusion. As a result, this protocol has an intrinsic limitation. Under practical RFB operating conditions, IEMs are exposed to an external electric field, so species crossover is governed not only by concentration‐driven diffusion but also by field‐driven electromigration. In other words, permeability parameters extracted under zero‐field conditions may not faithfully capture crossover behavior under realistic operating conditions. Accordingly, electric‐field‐assisted permeability characterization is required to provide a more representative assessment of transmembrane transport under RFB conditions.

Under an applied electric field, ion transport in charged channels follows the Nernst–Planck framework as discussed above, where diffusion and electric field driven migration jointly govern the rate of species transfer across the membrane. Experimentally, a straightforward strategy is to upgrade the conventional H‐type diffusion cell into an electrochemically biased two‐compartment configuration, the membrane separates two well‐stirred electrolyte reservoirs, a constant potential difference Δϕ is imposed across the membrane via electrodes, and the time evolution of the receiving‐side concentration ***C***_***t***_is simultaneously quantified (e.g., by UV–vis/ICP). The permeation behavior can still be expressed in terms of the transmembrane flux **J**, which is linked to the concentration evolution through a mass balance:

(6) $$V\;\frac{dC_{t}}{\mathrm{dt}}=A\cdot J$$

Under common approximations a membrane thickness of ***L***, and approximately linear concentration and potential gradients along the thickness direction, **Δ** ***c***_***i***_ ≈ (***C***_***t***_ − ***C***_0_)/***L***, the flux can be expressed as the sum of a diffusion term and an electromigration term:

(7) $$J\;=\frac{D_{i}}{L}\;\left(C_{0}-C_{t}\right)+\frac{z_{i}F}{\mathrm{RT}}D_{i}c_{i}\Delta \phi$$

Compared with the zero‐field case in which the diffusive flux is driven solely by (***C***_0_ ***C***_***t***_), the electromigration contribution introduced by Δϕ under an applied electric field can markedly alter the transmembrane transport rate, thereby rendering the permeability characterization more representative for practical operating conditions.

### 4.5 Water Uptake and Swelling Ratio

The pronounced hydrophilicity of polymer membranes often leads to considerable swelling in aqueous electrolytes, which can compromise their dimensional stability and ion selectivity [112]. To ensure membrane reliability under operational conditions, quantitative evaluation of water uptake (WU) and swelling ratio (SR) becomes essential.

(8) $$\mathrm{WU}\;=\frac{W_{\mathrm{wet}}-W_{\mathrm{dry}}}{W_{\mathrm{dry}}}\;\times 100\%$$

(9) $$\mathrm{SR}\;=\frac{L_{\mathrm{wet}}-L_{\mathrm{dry}}}{L_{\mathrm{dry}}}\;\times 100\%$$

where ***W***_***wet***_ and ***W***_***dry***_ are the weights of the wet membranes and dry membranes, respectively. ***L***_***wet***_ and ***L***_***dry***_ are the lengths of the wet membranes and dry membranes, respectively.

### 4.6 Morphology and Molecular Structure

The morphological characteristics of the membranes are typically examined using scanning electron microscopy (SEM), which provides direct visualization of surface and cross‐sectional architectures. Complementary chemical characterization can be achieved through Fourier‐transform infrared spectroscopy (FTIR) and nuclear magnetic resonance (NMR), both of which are powerful tools for identifying functional groups, elucidating bonding environments, while quantitative determination of the degree of sulfonation requires an appropriate calibrated method. Together, these techniques are indispensable for evaluating membrane integrity, probing microphase separation and spatially resolved structural feature, and detecting potential structural defects that may compromise ion transport pathways and ultimately impair battery performance.

### 4.7 The Cell Performance Evolution

The impact of membranes on RFB performance is typically assessed through galvanostatic charge‐discharge cycling, which provides key metrics including Coulombic efficiency (CE), voltage efficiency (VE), and energy efficiency (EE). Ideal membranes should enable minimal polarization, high capacity retention, and stable voltage plateaus during cycling [24].

(10) $$\mathrm{CE}\;=\frac{\int_{0}^{t_{d}}I_{d}\left(t\right)\mathrm{dt}}{\int_{0}^{t_{c}I_{c}\left(t\right)\mathrm{dt}}}\;\times 100\%$$

(11) $$\mathrm{VE}\;=\frac{\int_{0}^{t_{d}}U_{d}\left(t\right)\mathrm{dt}}{\int_{0}^{t_{c}}U_{d}\left(t\right)\mathrm{dt}}\;\times 100\%$$

(12) $$\mathrm{EE}\;=\mathrm{CE}\times \mathrm{VE}\;=\frac{\int_{0}^{t_{d}}U_{d}\left(t\right)\mathrm{dt}\cdot I_{d}\left(t\right)\mathrm{dt}}{\int_{0}^{t_{c}}U_{d}\left(t\right)\mathrm{dt}\cdot I_{c}\left(t\right)\mathrm{dt}}\;\times 100\%$$

where ***t***_***d***_ and ***t***_***c***_ denote the discharge time and charge time of the battery respectively. ***I***_***d***_ and ***I***_***c***_ denote the current during charge and discharge of the battery, respectively. ***U***_***d***_and Uc denotes the voltage during charge and discharge of the battery, respectively.

### 4.8 Performance Metrics as Benchmarks and Remaining Gaps

PFSA membranes, typified by Nafion 117, have long dominated the commercial landscape of RFB membrane owing to their outstanding comprehensive performance [24]. These membranes combine high mechanical robustness (tensile strength >30 MPa), excellent ionic conductivity, and substantial water uptake (∼40 wt.%), while also exhibiting remarkable chemical stability and cycling durability (>5,000 cycles) under harsh operating conditions. Performance radar plots consistently highlight this balanced property profile. Nevertheless, large‐scale application of PFSA remains constrained by three critical limitations: (i) prohibitively high cost (>$1500 m^−2^), (ii) excessive vanadium ion permeability (∼5 × 10^−7^ cm^2^ s^−1^), and (iii) insufficient proton selectivity (<102), which collectively accelerates capacity decay (0.3%–0.8% per cycle). To alleviate these limitations, non‐fluorinated sulfonated aromatic polymers, such as SPEEK, SPES, SPI, SPBI and others [112, 113, 114, 115, 116, 117, 118, 119, 120, 121], have been widely investigated as cost‐effective and chemically tunable alternatives. However, their practical implementation is often hindered by the intrinsic conductivity–swelling–selectivity coupling. Typically, increasing sulfonation density to boost conductivity frequently leads to high water uptake and pronounced dimensional swelling, which weakens mechanical integrity and can aggravate crossover, while overly rigid backbones or aggressive sulfonation conditions may also compromise processability and oxidative stability. To address these challenges, recent efforts across both commercial and emerging IEMs platforms have increasingly emphasized sulfonation‐enabled control of membrane chemistry and morphology, focusing on regulating the –SO_3_H content and its spatial distribution as well as the resulting ionic‐domain size, connectivity, and swelling response. In parallel, interfacial engineering and composite reinforcement are being employed to constrain swelling, stabilize transport channels, and suppress crossover, thereby improving durability and cost‐effectiveness without sacrificing conductivity. Altogether, these directions aim to move membrane performance beyond the conventional conductivity–selectivity trade‐off. At the same time, they seek to retain long‐term stability and scalability for large‐scale aqueous energy‐storage deployment [24, 122]. Together, these measurements provide the basis for evaluating the structure–property relationships of the representative membrane systems discussed in Section 5.

## 5 Design and Application of Sulfonated Polymeric Membranes for RFBs

Membrane requirements depend on both the ions needed to maintain charge balance and the redox‐active species that should remain in their respective half‐cells. In VRFBs, low membrane resistance must be balanced against crossover of vanadium species and stability in acidic electrolytes across the operating states of charge. In zinc‐based batteries, the required ion transport must also be considered alongside zinc deposition and stripping, wet mechanical integrity, and the species targeted for crossover suppression; these species differ among zinc–bromine and other zinc chemistries [123]. In aqueous organic RFBs, the size, charge, and chemical state of redox‐active molecules may depend on pH and state of charge, affecting partitioning, adsorption, and crossover. Thus, –SO_3_H‐containing membranes should be evaluated in their electrolyte‐equilibrated ion‐exchange state: the dominant charge carrier cannot be inferred from the acid group alone (Figure 7).

**FIGURE 7:**
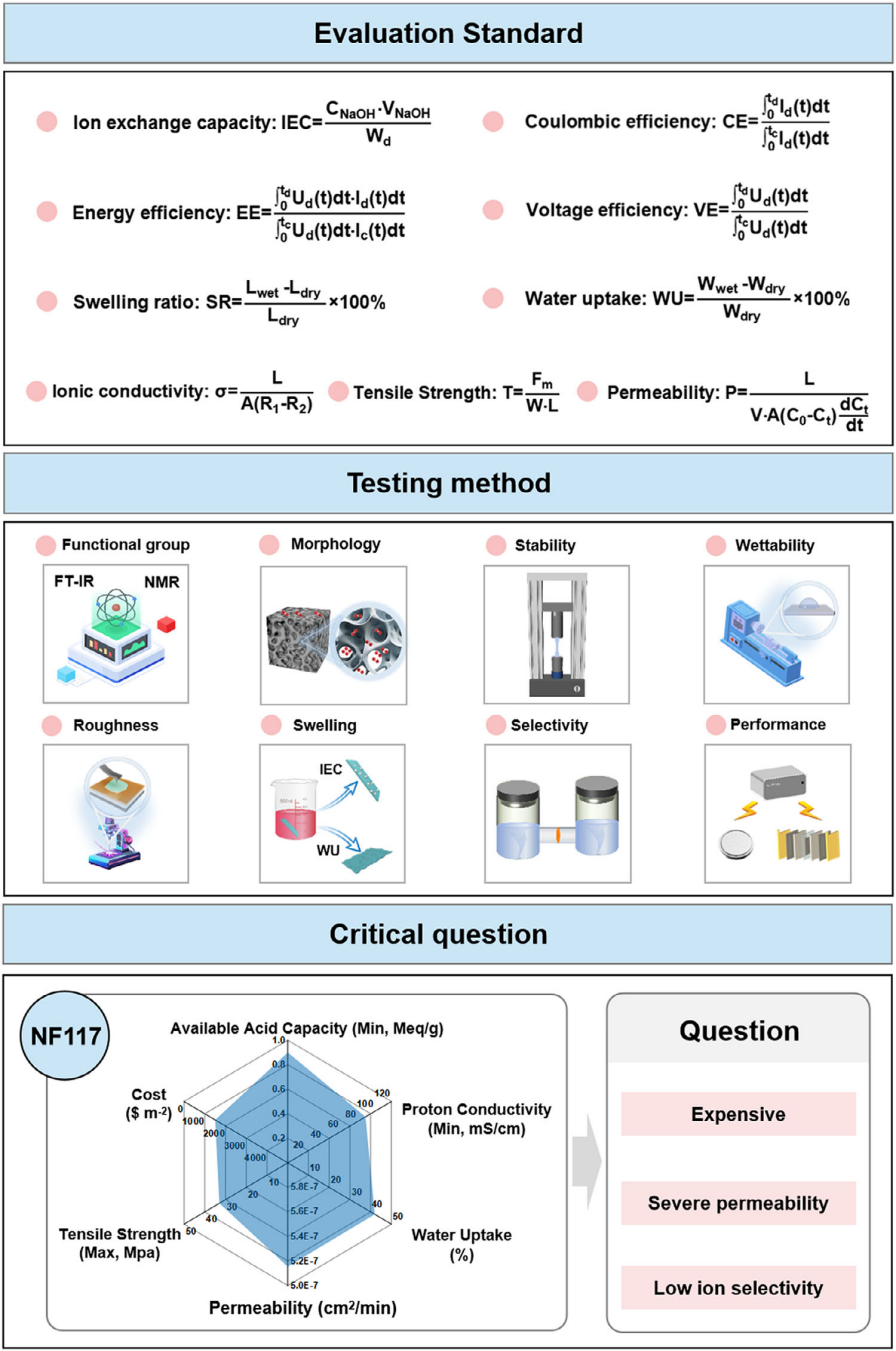
Membrane evaluation criteria, performance benchmarks for the widely used Nafion 117 membrane, and representative membrane testing methods.

### 5.1 Perfluorosulfonated Flexible Membranes

Fluorinated polymer membranes have found widespread application in emerging energy systems, pervaporation, and gas separation, owing to their outstanding chemical stability, mechanical flexibility, and tunable ion transport characteristics. Among them, PFSA polymer (exemplified by DuPont's Nafion) has become the most extensively investigated class of proton‐conductive membranes, primarily due to their unique microphase‐separated architecture comprising interpenetrating hydrophilic and hydrophobic domains. Since their introduction in the 1970s, Nafion membranes have established a benchmark for proton exchange materials by constructing efficient proton‐conducting channels through ionic clusters of –SO_3_H groups. The synergy between their highly crystalline polytetrafluoroethylene backbone and moderately flexible perfluorovinyl ether side chains provide an exceptional balance between mechanical robustness and ion transport. In particular, the chemically inert PTFE framework ensures structural stability. In contrast, the hydrated sulfonated side chains assemble into dynamic, interconnected networks that facilitate rapid proton conduction. This multiscale structural design paradigm has not only underpinned Nafion's long‐standing success in RFBs, but also informed subsequent innovations in functionalized fluorinated membranes [124].

Recent advances in PFSA membranes for RFBs have focused on controlling ionic domain microstructure and engineering composites to reconcile the trade‐offs between conductivity and selectivity. Zhai et al. [125]. implemented a supramolecular “repair” strategy in Nafion, reducing the size of ionic microphase to ∼1 nm via synergistic non‐covalent interactions among Nafion, fluorinated block copolymer, and polyoxometalates, thereby promising a precise nanoarchitectural tuning (Figure 8a). Kim et al. [126] achieved bottom‐up control by pre‐aligning ionomers in aqueous substrates, yielding highly ordered ionic channels with ∼55% crystallinity and optimized transport pathways (Figure 8b). Hwang et al. [127] controlled the area stretching ratio γ by swelling Nafion 115 in water at 30°C, 60°C, and 90°C followed by constrained the swelling and annealing at 130°C, and by mechanically biaxially stretching Nafion 117°C at 160°C at an elongation rate of 1 mm s^−1^; they found that thickness independent proton conductivity and vanadium permeability decrease markedly up to γ ≈ 1.25 and then level off, stabilizing PFSA performance at high current densities. (Figure 8c). To enhance selectivity while mitigating swelling, Li et al. [128] introduced hydrophobic PVDF with high‐surface‐area Ketjen black into Nafion. The resulting electrostatic/structural reinforcement suppressed solvent uptake and improved ion sieving (Figure 8d). Processing can also reshape the conductivity–selectivity balance. Peng et al. [129] found that boiling pretreatment reduces the Donnan potential, increases solution uptake, and accelerates proton transport, boosting conductivity but simultaneously raising vanadium permeability. The observed trade‐off is governed by (i) the absorption capacity of the membrane for solvents and (ii) the intrinsic mobility of absorbed species within the matrix (Figure 8e). Dimensional and orientational control further promotes selectivity. Lee et al. [130] developed PFSA membranes with highly aligned ionic channels through molecular arrangement at the air/water interface. The well‐ordered ionic architecture remarkably suppressed vanadium ion crossover, achieving a 500‐fold enhancement in ion selectivity compared to conventional PFSA membranes (Figure 8f). So et al. [58] applied uniaxially stretched PFSA membranes to VRFBs, effectively suppressing cross‐membranes penetration of vanadium ions (Figure 8g). Vijayakumar et al. [131] systematically tuned PFSA equivalent weight to regulate sulfonate density and ionic domain geometry, showing that higher equivalent weight (EW) tightens hydrated transport pathways and markedly suppresses vanadium crossover at the expense of some proton conductivity. By reducing membrane thickness, they compensated the added ohmic penalty and achieved a thin, high EW PFSA that combines low crossover with low resistance, thereby improving VRFB cycling efficiency. (Figure 8h). Collectively, these studies show that supramolecular confinement, interfacial self‐assembly, strain‐induced alignment, and composite reinforcement are complementary approaches for programming PFSA microstructure, thereby tuning proton transport and ion crossover.

**FIGURE 8:**
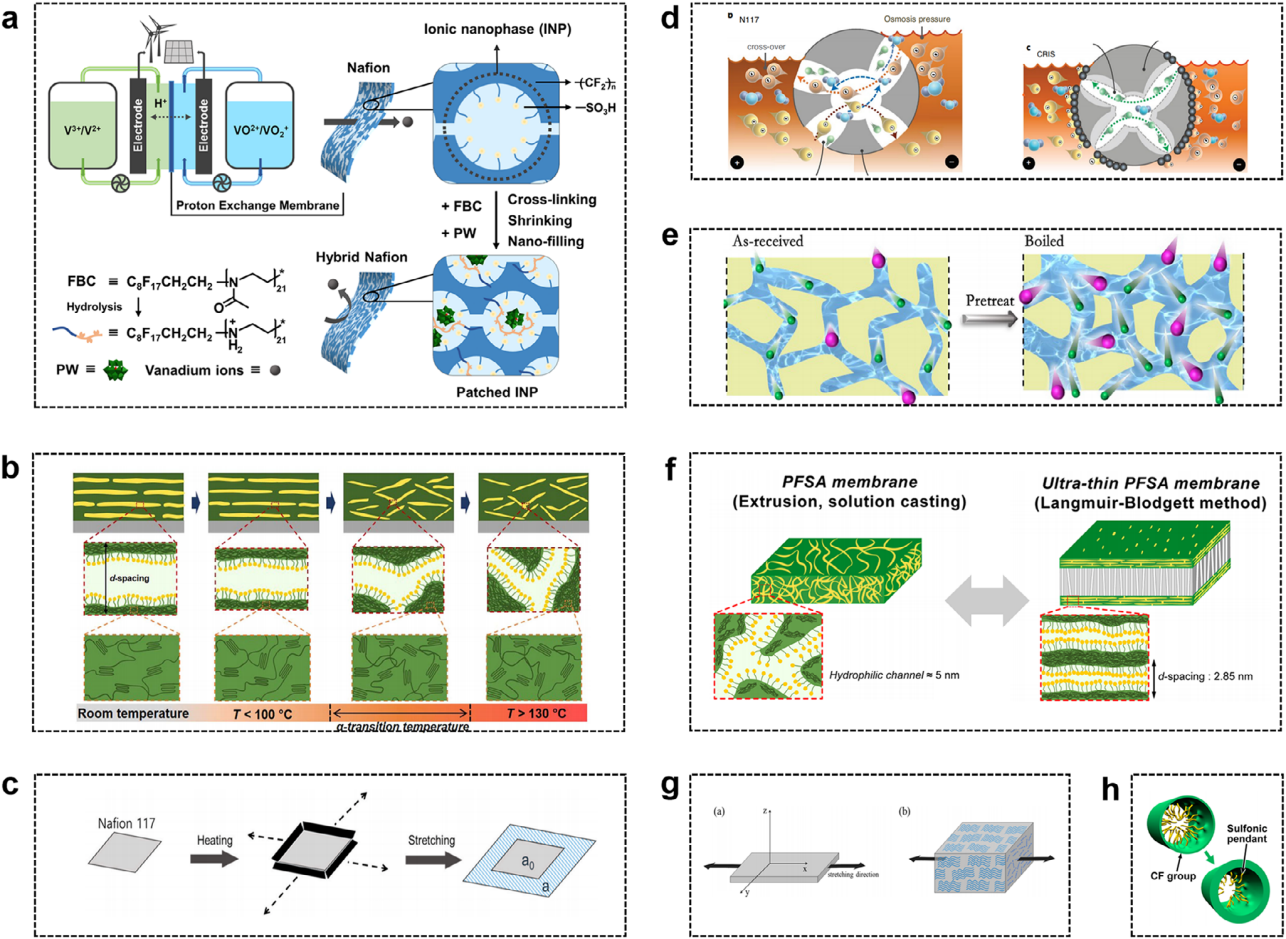
(a)Nanostructure modulation. Reproduced with permission [125]. Copyright 2023, American Chemical Society. (b) Ion transport pathway optimization. Reproduced with permission [126]. Copyright 2023 Royal Society of Chemistry. (c) Biaxial stretching. Reproduced with permission [127]. Copyright 2021 Elsevier. (d) Incorporating Ketjen black. Reproduced with permission [128]. Copyright 2021 Springer Nature. (e) Boiling treatment. Reproduced with permission [129]. Copyright 2023 American Chemical Society. (f) Aligning ion channels Reproduced with permission [130], Copyright 2021 American Chemical Society. (g) Uniaxial stretching. Reproduced with permission [58]. Copyright 2018 American Chemical Society. h) Morphology modification. Reproduced with permission [131]. Copyright 2018 American Chemical Society.

Recent studies highlight composite and supramolecular routes for programming PFSA microstructure as well as interfacial chemistry to decouple conductivity from crossover. Ye et al. [51] dispersed functionalized nanowires uniformly within PFSA and laminated an ultrathin PTFE skin onto the membrane surface, yielding a sandwich structure that prolongs vanadium diffusion paths. Meanwhile, surface groups on the rigid nanowires generate additional proton‐conduction sites (Figure 9a). Han et al. [132] embedded zwitterionic‐functionalized silica into Nafion. Sulfonated silica surfaces boosted ionic conductivity and simultaneously restricted active‐species crossover, improving voltage efficiency in ZnRFBs (Figure 9b). Extending this concept, Ye et al. [52] incorporated superhydrophilic TiO_2_ nanotubes into PFSA membranes. The sulfonic acid groups preferentially adsorb onto the nanotube and become more uniformly distributed along the tube walls, while the nanotube scaffold increases transport tortuosity for redox species. Together, these effects suppress crossover and improve ionic selectivity (Figure 9c). Using a similar approach, the same team [133] constructed an interlayered PFSA hybrid by embedding GO nanosheets decorated with in situ‐grown hydrophilic WO_3_ nanoparticles within a reinforced PTFE thin layer, thereby introducing a mechanically robust barrier that mitigates swelling and reduces species crossover. GO serves as a physical barrier that increases the tortuosity for vanadium species, thereby suppressing crossover, while WO_3_ nanoparticles grown on GO introduce proton‐active sites that facilitate proton conduction and reduce the interfacial transport penalty. Together, the GO–WO_3_ heterostructure combines effective vanadium rejection ability with high proton transport efficiency (Figure 9d). Hybridization with inorganic ion conductors can also strengthen mechanics and reconstruct transport pathways. Wang et al. [134] prepared Nafion/PVDF/LLZTO membranes that exhibit an ionic conductivity of 0.29 mS cm^−1^ and superior mechanical performance. Computational analysis confirmed the formation of complementary Li^+^ migration pathways and dehydrogenation sites at polymer–ceramic interfaces (Figure 9e). He et al. [135] developed a supramolecular co‐assembly strategy by introducing fluoroalkyl‐grafted polyoxometalates (POMs) nanoclusters as additives. The fluoroalkyl chains anchor the clusters into the perfluorinated matrix, while the inorganic POMs cores preferentially reside in the ionic domains, supplying additional proton‐transfer sites and nanoscale steric hindrance that improves both conductivity and selectivity (Figure 9f). Lee et al. [136] synthesized a sub‐20 nm composite film with PFSA‐grafted GO, where the grafted ionomer enhances mechanical robustness and establishes efficient proton pathways, delivering higher energy efficiency than commercial references (Figure 9g).

**FIGURE 9:**
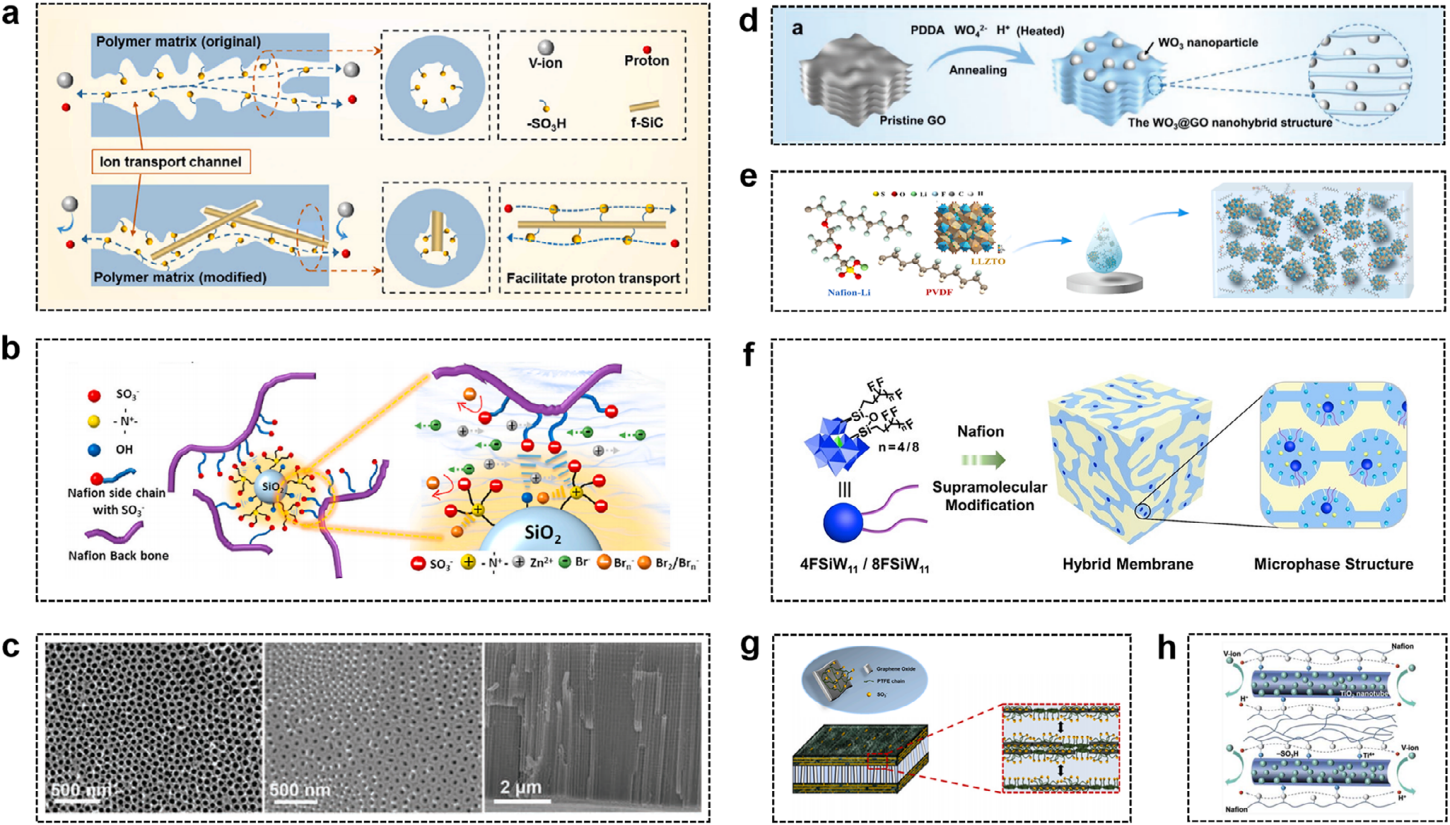
(a) Functionalized doped nanowires. Reproduced with permission [51]. copyright 2022 Elsevier. (b) Functionalized doped SiO_2_. Reproduced with permission [132]. Copyright 2022 Elsevier. (c) Hydrophilic doped TiO_2_ nanotubes. Reproduced with permission [52]. Copyright 2020 Wiley‐VCH Verlag. (d) Anchoring WO_3_ on graphene oxide. Reproduced with permission [133]. Copyright 2022 Wiley‐VCH Verlag. (e) Using inorganic ceramics as fillers. Reproduced with permission [134]. Copyright 2023 Elsevier. (f) Grafted polyoxometalate nanoclusters. Reproduced with permission [135]. Copyright 2024 John Wiley and Sons. (g) Grafted graphene oxide. Reproduced with permission [136]. Copyright 2023 Elsevier.

For fluorinated flexible membranes, exemplified by PFSA, intrinsic microphase separation provides the structural basis for four widely adopted design strategies. (i) tortuosity/barrier engineering (PTFE skins, GO sheets, nanotubes) to lengthen and constrain vanadium diffusion; (ii) interfacial proton transport engineering (sulfonated silica, WO_3_, POM clusters) that densifies hopping sites while maintaining Donnan exclusion; (iii) supramolecular co‐assembly to immobilize functional fillers within PFSA ionic domains without disrupting percolated proton pathways; and (iv) mechanical co‐reinforcement with ceramic/carbon frameworks to sustain ultra‐thin, processable formats. The guiding principle is to harness PFSA's tunable microstructure, via orientation, stretching, interfacial self‐assembly, and hybridization, to simultaneously preserve continuous proton networks and impose anisotropic, tortuous pathways for multivalent ions. Similar fillers need not produce the same loading‐dependent response: dispersion, aggregation, interfacial compatibility, and the resulting hydrated morphology determine whether added filler creates useful transport pathways or introduces defects.

These strategies leverage the hallmark advantages of fluorinated flexible membranes, such as exceptional chemical/thermal robustness in harsh electrochemical environments, high processability (thin films, alignment), and broad compatibility with inorganic and supramolecular modifiers. Remaining challenges include filler–ionomer interfacial aging under long‐term cycling, water‐uptake management at high IEC, crossover suppression at reduced thickness, and scalable, reproducible processing that preserves nanoscale order. At high filler loading, aggregation may increase brittleness, while repeated electrolyte uptake and swelling may promote interfacial debonding or filler loss during cycling. Achieving these targets will translate PFSA's industrial maturity and structural tunability into next‐generation, cost‐effective separators for RFBs.

### 5.2 Perfluorosulfonated Rigid Membranes

Recent work has leveraged rigid, crystallographically ordered scaffolds, in which chemically inert perfluorinated ionomers are integrated into sub‐nanometer channel architectures, to improve selectivity while retaining efficient ion transport. Pang et al. [137] reported a self‐supporting structural membrane by interweaving a sulfonated covalent organic framework (SCOF) with Nafion chains. In this design, a wedge‐and‐tenon like interlocking between the SCOF scaffold and infiltrated Nafion chains mechanically stabilizes in a continuous network of densely packed –SO_3_H sites, facilitating efficient proton transport via the Grotthuss mechanism while suppressing swelling. Concurrently, hydrogen‐bonding interactions confine the flexible PFSA chains within the SCOF pores, reducing the effective pore size from ∼15.0 to 5–10 Å. This size reduction significantly enhances proton–vanadium ion selectivity (Figure 10a). Separately, Han et al. [138] tackled crossover in Zn–Br systems by developing a composite membrane embedded with amidated/sulfonated UiO‐66. The (Metal–Organic Framework) MOF cages and amine functionalities selectively trap Br^−^ to suppress crossover. Meanwhile, the tuned hydrophilic domains establish well‐connected water channels for balancing Zn^2+^ transport with bromide retention (Figure 10b). Here, maintaining Zn^2+^ transport and limiting bromine‐species crossover are distinct membrane requirements; the results should not be interpreted using the vanadium‐crossover criterion alone. Ji et al. [57] constructed a hierarchical membrane by (i) synthesizing 2D BN nanosheets, (ii) grafting sulfonic acid groups onto BN nanosheets using Nafion as a sulfonated side‐chain precursor to raise the IEC, and (iii) incorporating the sheets into a porous PEI matrix, thereby alleviating ion‐transport limitations in VRFBs through ordered, acid‐functionalized pathways (Figure 10c). By addressing dendrite growth in zinc batteries, Xia et al. [78] introduced rigid Zn–N suspended ionic arrays into a thin film. This incorporation improved puncture resistance and mechanical strength, effectively blocking Zn dendrites growth and enabling stable cycling for 550 cycles (Figure 10d). These zinc‐based examples also illustrate the limits of transferring membrane design rules directly from VRFBs. In VRFBs, vanadium crossover is undesirable, whereas Zn^2+^ transport is needed in many aqueous zinc batteries, including zinc‐based flow systems. The useful feature is therefore not a general barrier to multivalent ions, but a suitable distribution of fixed charges and hydrated pathways for the ions required by the particular cell. Sulfonate‐containing interfaces may help regulate local Zn^2+^ distribution, although strong Zn^2+^ association could impede its mobility. Electrolyte compatibility, water uptake, wet mechanical integrity, and resistance to zinc penetration must also be considered. Whether improved ion distribution leads to more uniform zinc deposition should be verified in the relevant cell rather than inferred from membrane conductivity alone.

**FIGURE 10:**
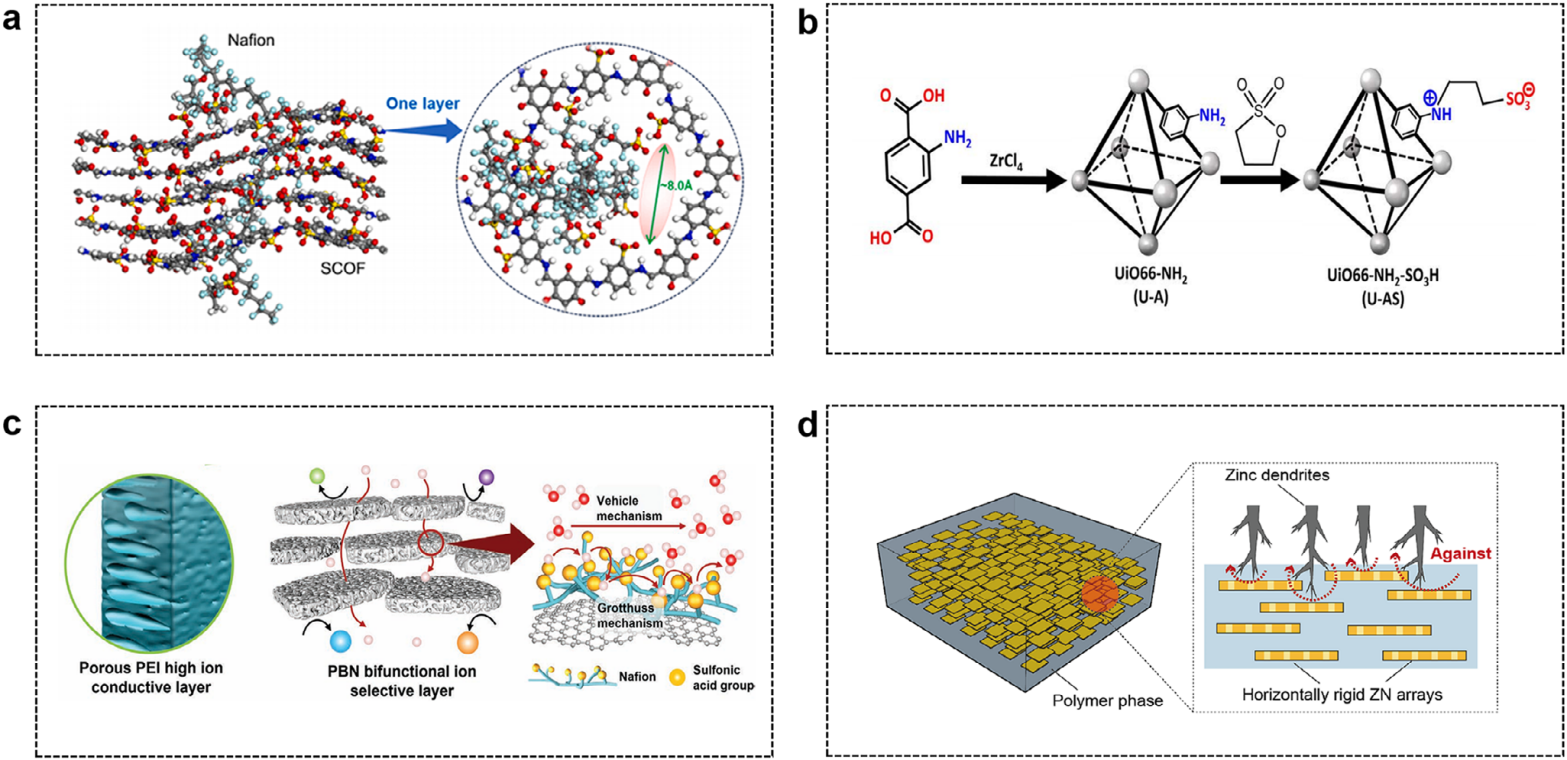
(a) Pore size reduction in COFs. Reproduced with permission [137]. Copyright 2024 Elsevier. (b) Hydrophilic channel engineering. Reproduced with permission [138]. Copyright 2024 Royal Society of Chemistry. (c) Integration of rigid zinc‐nitrogen suspended ion arrays. Reproduced with permission [57]. Copyright 2024 Elsevier. (d) Mitigating ion transport limitations. Reproduced with permission [78]. Copyright 2023 John Wiley and Sons.

Perfluorosulfonated rigid membranes and their hybrids, which incorporate PFSA within ordered, rigid frameworks, provide a distinct set of advantages: (i) dimensional stability and ultralow swelling; (ii) exceptional chemical stability of the perfluorinated backbone in strongly acidic and oxidizing electrolytes; (iii) crystallographically defined –SO_3_H arrays that support Grotthuss‐dominated proton percolation; and (iv) size‐ and Donnan‐exclusion effects via sub‐nanometer pores and ion selectivity that suppress multivalent or halide crossover. By integrating PFSA into rigid COFs/MOFs/2D BN scaffolds, either by physically anchoring PFSA chains within their pores or by chemically grafting sulfonated moieties onto the scaffold, these membranes simultaneously maintain high conductivity and sharpen selectivity. Enhanced mechanical toughness further enables thinner, lower‐resistance membrane configurations. Thus, this rigid–perfluorosulfonated design represents a compelling route toward durable, application‐tuned separators for VRFBs and ZnRFBs.

### 5.3 Non‐Perfluorosulfonated Flexible Membranes

Non‐perfluorinated sulfonated polymers serve as a tunable platform for PFSA‐free separators, allowing controlled sulfonation, tailored backbone/side‐chain design, and composite reinforcement. Ding et al. [139] introduced 5‐sulfoisophthalic acid monosodium salt into the PBI backbone, enabling precise control of the DS. By tuning DS, the hydrophilicity and ionic conductivity was increased while identifying an optimal sulfonation window could balance ionic conductivity against crossover for RFB operation. Zhang et al. [140] prepared sulfonated polysulfone (SPSF) membranes with varied DS, determining that a DS of 62% provided the best balance between ionic conductivity and vanadium blocking in VRFBs (Figure 11b). Liu et al. [141] designed sulfonated polybenzimidazole (SPBI) membranes incorporating flexible diether segments. (2‐sulfo‐1,4‐phenylene)bis(oxy)dibenzoic acid was used as a reactive diether monomer to improve the solubility and promote the microphase separation. Copolymerization with 5‐sulfoisophthalic acid monosodium salt yielded SPBI with IEC of 2.08 mmol g^−^^1^, an area resistance of 0.21 Ω cm^2^, and vanadium‐ion permeability of 3.36 × 10^−9^ cm^2^ s^−1^, creating excellent selectivity in VRFBs (Figure 11c). Xu et al. [142] blended long‐side‐chain sulfonated poly(triphenylene) (SPTP) with PBI to form composites. Vanadium transport is suppressed via Donnan exclusion, reduced acid uptake, and low swelling, while an ether‐free backbone, electrostatic repulsion, and PBI reinforcement enhance mechanical integrity and chemical durability (Figure 11d). From a sustainability perspective, Lander et al. [143] fabricated dense, tightly cross‐linked sulfonated cellulose membranes with limited water swelling and high aqueous stability in ORFBs. These sustainable membranes surpassed Nafion 115 in cycling stability for ORFBs (Figure 11e). This result was obtained in the reported aqueous ORFB and should not be taken as evidence of durability in strongly acidic, oxidizing vanadium electrolytes. For cellulose‐based membranes, wet strength, swelling, dimensional stability, and retention of ionic functionality need to be assessed in the intended electrolyte. Sulfonate groups and sulfate esters should also be distinguished because their chemical linkages differ. Yang et al. [144] grafted sodium 2‐bromoethanesulfonate or 1,4‐butanesultone onto ether‐free poly(terphenylene piperidinium) matrices. The resulting membranes, which exhibited low area resistance and high proton permeability while markedly suppressing vanadium crossover, consequently delivered high ion selectivity (Figure 11f).

**FIGURE 11:**
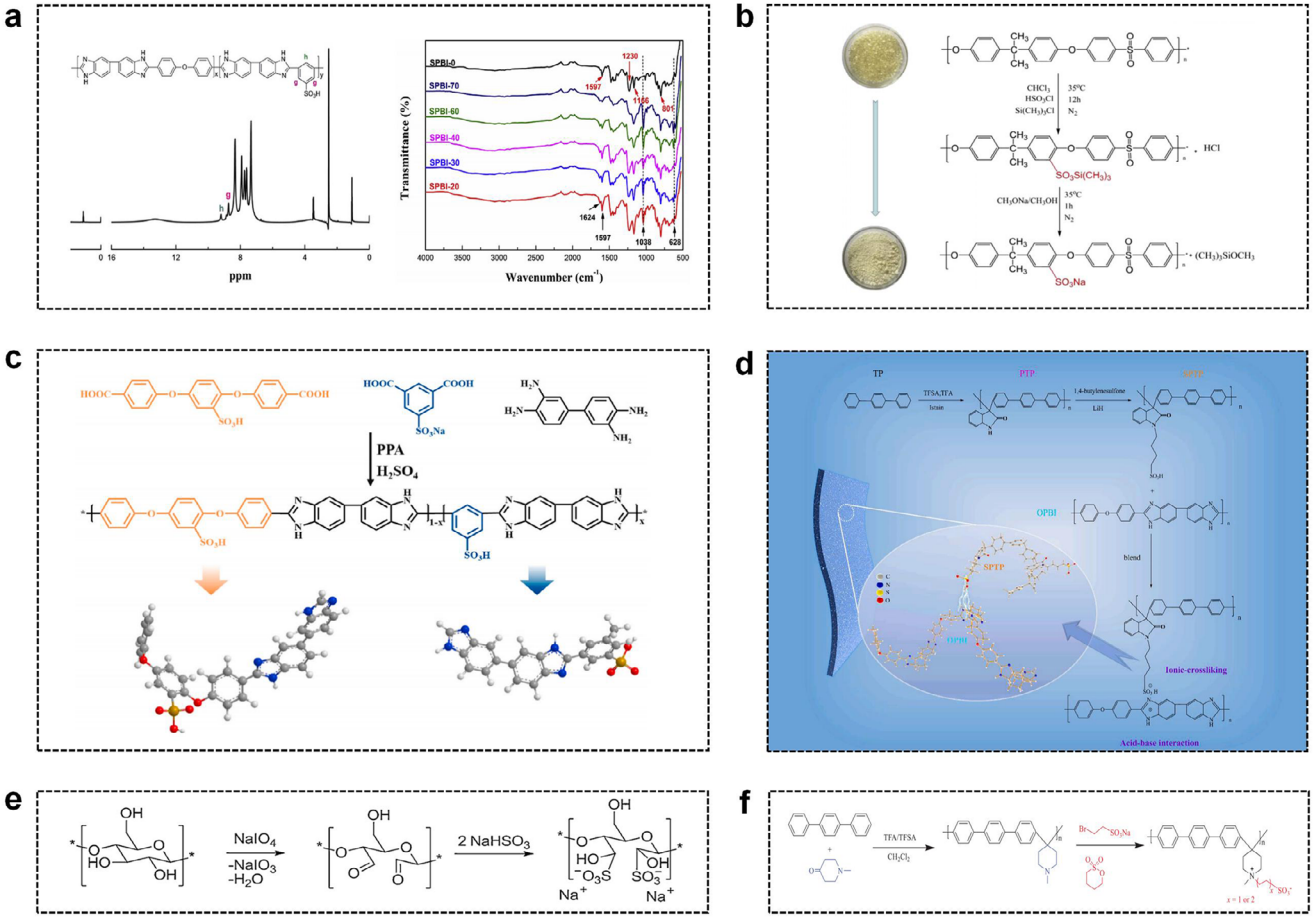
(a) Enhancing the hydrophilicity and ionic conductivity of PBI. Reproduced with permission [139]. Copyright 2019 Elsevier. (b) Controlling DS. Reproduced with permission [140]. Copyright 2019 Elsevier. (c) Promoting microphase separation. Reproduced with permission [141]. Copyright 2019 Elsevier. (d) Ether‐free backbones enhance stability. Reproduced with permission [142]. Copyright 2022 Elsevier. (e) Green organic materials also enable energy storage. Reproduced with permission [143]. Copyright 2021 John Wiley and Sons. (f) Ether‐free backbones enhance selectivity. Reproduced with permission [144]. Copyright 2024 Elsevier.

Building on microstructural engineering, Zhou et al. [145] fabricated PES membranes via ionic liquid–induced phase separation. These membranes combine an optimized porous microstructure with robust mechanical integrity, yielding an order‐of‐magnitude lower vanadium permeability than Nafion 212 while maintaining σ above 20 mS cm^−1^. In VRFBs, this combination of low permeability and high conductivity yielded EE comparable to Nafion 212 but a higher CE (Figure 12a). Santos et al. [146] converted lignin into sulfonated nanoparticles (NanoSL) and incorporated them into a polyvinyl alcohol (PVOH) matrix. Membranes with the highest NanoSL loading exhibited both high IEC and excellent conductivity, highlighting the potential of sustainable biomaterials for advanced membrane development (Figure 12b). However, lignin feedstock and processing can change molecular weight, functional‐group content, and compatibility with the host polymer. Higher sulfonated‐lignin loading should therefore be assessed not only through IEC and conductivity, but also through water uptake, wet mechanical properties, component retention, and batch reproducibility. Compared with PFSA or sulfonated aromatic membranes, biomass‐derived candidates require evaluation under matched electrolyte and test conditions before conclusions about transport or durability can be generalized. Bui et al. [147] fabricated multisulfonated polybenzimidazole (MS‐PBI) membranes by precisely controlling solvent residue content, ion‐exchange state, and membranes thickness. Their systematic study showed that fluorine‐free SPBI outperformed other variants in VRFBs, delivering >99% CE and 92.2% EE at 80 mA cm^−2^, together with remarkable long‐term durability (Figure 12c). Building on composite design for bromine crossover suppression, Duraisamy et al. [148] incorporated tunable WO_3_@GO loadings into SPEEK, and found that WO_3_‐decorated GO, owing to enhanced hydrophilicity and uniform dispersion, established strong interactions with –SO_3_H groups. The resulting films combined superior mechanical stability and ionic conductivity with effective suppression of bromine crossover, thereby improving Zn–Br RFB performance (Figure 12d). By extending this filler–polymer coupling paradigm, Beydaghi et al. [149] synthesized 2D NbS_2_ nanosheets, functionalized them with 3‐mercapto‐1‐propanesulfonic acid, and embedded the sulfonated (S‐NbS_2_) sheets within the SPEEK. This increased the ionic conductivity from 45.82 to 94.35 mS cm^−1^. Hydrogen bond‐mediated matrix densification, together with with physical obstruction, effectively impeded vanadium species diffusion (Figure 12e). Mukhopadhyay et al. [150] fabricated PVDF‐HFP/cellulose‐nanocrystal (CNC) composites via solution casting. The hydrophobic PVDF‐HFP backbone mitigated electrolyte crossover, while the abundant, low‐cost CNC provides –OH rich pathways that facilitate proton transport. High CNC crystallinity preserved mechanical and chemical stability in acidic electrolytes, generating flexible, strong, and highly selective membranes amenable to industrial scale‐up (Figure 12f). Yuan et al. [151] demonstrated pilot‐scale synthesis and roll‐to‐roll production of SPEEK membranes, showcasing their high OH^−^ conductivity and chemical stability in kilowatt‐scale alkaline RFBs. The membranes were integrated into a flow battery stack with a power output of 4,000 W, achieving an energy efficiency of 85.5% at 80 mA cm^−2^ and stable operation for over 800 h. Notably, this system exhibited significantly reduced costs compared to Nafion‐based counterparts. This work represents a critical step toward cost‐effective hydrocarbon membranes into to large‐scale grid energy storage applications (Figure 12g).

**FIGURE 12:**
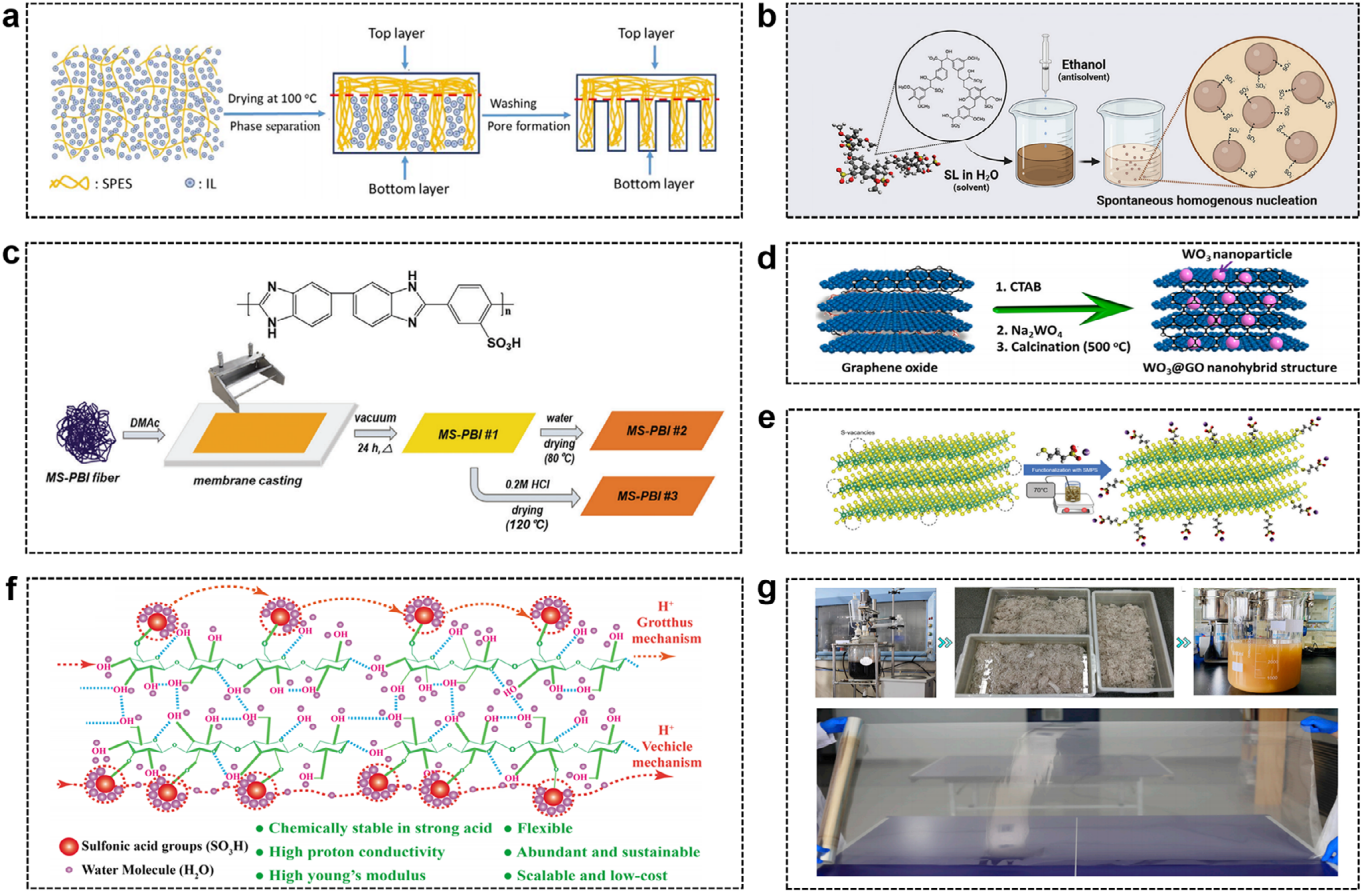
(a) Developing SPES membranes via ionic liquid‐induced phase separation. Reproduced with permission [145]. Copyright 2020 Elsevier. (b) Bio‐based materials show great potential. Reproduced with permission [146]. Copyright 2024 John Wiley and Sons. (c) Precisely controlling solvent residue content, ion‐exchange form, and membrane thickness to tailor membrane properties. Reproduced with permission [147]. Copyright 2024 John Wiley and Sons. (d) Doped WO_3_@GO. Reproduced with permission [148]. Copyright 2024 Elsevier. (e) Sulfonated doped NbS_2_. Reproduced with permission [149]. Copyright 2022 Royal Society Chemistry (f) Doped CNCs. Reproduced with permission [150]. Copyright 2019 American Chemical Society. (g) Pilot‐scale synthesis and roll‐to‐roll production. Reproduced with permission [151]. Copyright 2022 Cell Press.

Collectively, these studies show that fluorine‐free sulfonated membranes can be optimized through several practical levers. First, the DS should be tuned to balance proton conductivity with active‐species crossover. The effect of increasing DS also depends on where the ionic groups are located and how the membrane swells in the intended electrolyte. Greater carrier transport can therefore coincide with either improved selectivity or increased active‐species crossover. Second, polymer backbone and side‐chain architecture can be tailored to form well‐connected ionic domains. Third, composites can combine Donnan exclusion and size sieving with effective swelling control. Looking ahead, improving chemical stability in strongly acidic and oxidizing electrolytes at high IEC, limiting swelling during cycling, and developing scalable syntheses with well‐controlled grafting and crosslinking will be essential for reproducible fluorine‐free membranes for VRFBs. ZnRFBs and ORFBs.

Recent work has examined bar coating, screen printing, and slot‐die coating of cellulose nanofibrils on kraft paper as potential manufacturing routes [152]. These coatings demonstrate the relevance of ink formulation and defect control, but use carboxymethylated cellulose rather than sulfonic‐acid‐functionalized cellulose; their short‐term transport results do not establish long‐term durability in an acidic flow‐battery stack.

### 5.4 Non‐Perfluorosulfonated Rigid Membranes

Non perfluorinated rigid membranes have emerged as a promising separator platform for aqueous RFBs because their shape‐persistent backbones and intrinsic microporosity can create ordered transport nanochannels. Such ordered channels are attractive for sustaining fast proton conduction while regulating ion sieving. However, in polymer‐based systems, achieving precise and reproducible control over nanochannel geometry and chemistry remains challenging. To address this, Xin et al. [62] embedded two acid‐stable Zr‐MOFs (MOF‐801 and MOF‐808)) with tunable pore architectures into a SPEEK. The smaller triangular windows of MOF‐801 provided molecular‐sieving ability that raised Coulombic efficiency, whereas the interconnected proton pathways of MOF‐808 established “proton highways” that improved voltage efficiency. This dual‐MOF composite synergistically boosted RFB performance (Figure 13a). Wu et al. [153] used sulfonated macromolecular chains to mediate the crystallization of a COF. The –SO_3_H groups helped guide COF crystallization during membrane formation. As a result, the framework developed a more continuous, well‐aligned network of acid‐rich nanochannels, enabling rapid, selective proton transport while suppressing organic crossover (Figure 13b). For seeking diffusion‐limited transport, Zuo et al. [64] constructed large‐area, free‐standing COF with rigidly confined ionic channels. Quasi‐frictionless ion flow originated from the combined effects of strong steric confinement and ion–framework interactions. Sulfonic‐acid functionalization was used to tune the pore chemistry. Simulations showed that the rigid SCTF‐BP (after force‐field aging) framework best preserved interconnected pores and resisted swelling, making it a strong candidate for ion‐selective membranes. (Figure 13c). To address polymer–filler compatibility, Xia et al. [86] introduced a PIM into highly sulfonated SPEEK (HDS‐SPEEK), where the rigid backbone structure of PIM effectively resolved the poor mechanical strength and vanadium resistance of HDS‐SPEEK. Experimental results demonstrated that with increasing the content of carboxyl‐grafted PIM, the tensile strength improved from 15.22 to 29.94 MPa, while vanadium permeability decreased from 4.4×10^−6^ to 1.6×10^−7^ cm^2^ min^−1^. The assembled VRFBs achieved a remarkable increase in Coulombic efficiency from 55% to 99% (Figure 13d). By using rigid porous fillers, Xu et al. [61] dispersed a sulfonated piperazine covalent triazine framework into SPEEK. Intrinsic nanochannels imparted pronounced Donnan exclusion, and N‐rich sub‐2nm pores formed proton highways. Moreover, externally grafted –SO_3_H further accelerated proton transfer. Simulations showed a substantially higher transmembrane energy barrier for vanadium ions than for hydrated protons, which explains the measured selectivity (Figure 13e). By introducing ion‐coordination motifs into the backbone, Zhu et al. [154] synthesized SPBA‐18‐x IEMs bearing anionic centers and crown‐ether units. Electrostatic interactions between DB18C6–H^+^ complexes and SO_3_^−^ sites organized quasi‐ordered ionic channels and a cooperative hydrophilic network, enabling efficient proton conduction with exceptionally low Fe^2+^ permeability (Figure 13f). Huang et al. [155] fabricated a rigid hierarchical ceramic composite with a sub‐10nm polyelectrolyte coating that pairs high selectivity with high conductivity. The architecture suppresses dendrite formation and, together with SPEEK‐derived sulfonate groups that facilitate ion transport, in concert with SPEEK‐derived sulfonates, supports extended cycle life and high areal capacity (Figure 13g).

**FIGURE 13:**
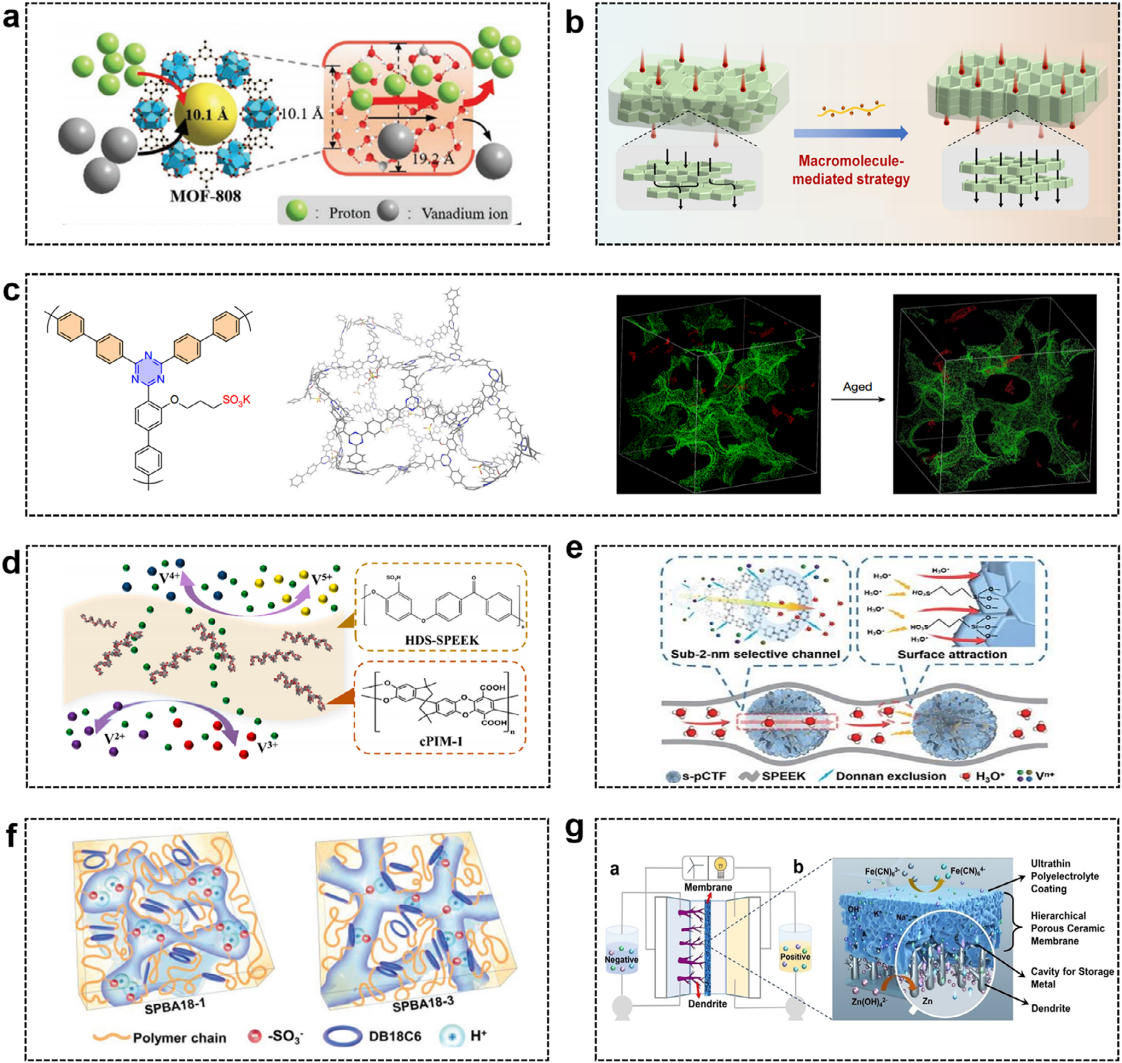
(a) Designing ordered nanochannels. Reproduced with permission [62]. Copyright 2021 John Wiley and Sons. (b) Sulfonated macromolecule incorporation strategy. Reproduced with permission [153]. Copyright 2023 John Wiley and Sons. (c) Constructing covalently bonded polymer frameworks with rigidly confined ion channels. Reproduced with permission [64]. Copyright 2023 Springer Nature. (d) Compatibility challenges between polymer and porous fillers. Reproduced with permission [86]. Copyright 2022 Elsevier. (e) Formation of hydrophilic networks via electrostatic interactions. Reproduced with permission [61]. Copyright 2023 John Wiley and Sons. (f) Developing sub‐10 nm polyelectrolyte coatings. Reproduced with permission [154]. Copyright 2023 John Wiley and Sons. (g) Porous ceramic composite membrane. Reproduced with permission [155]. Copyright 2024 John Wiley and Sons.

Across these approaches, the common goal is to introduce rigid, well‐defined transport channels with tunable chemistry. These channels preserve continuous proton conduction. At the same time, multivalent ions and redox‐active molecules are rejected through size sieving and Donnan effects. These gains are achieved without sacrificing dimensional stability or mechanical reliability. This logic aims to construct rigid, ordered, and chemically tailored nanochannels that establish continuous proton‑conduction pathways while enforcing discrimination based on size and Donnan exclusion against multivalent or organic species. Simultaneously, swelling and mechanical integrity are tightly controlled. As a result, these rigidity‑enhanced non‑fluorinated membranes can achieve both high selectivity and high conductivity in RFB environments. Rigidity or a small nominal pore size alone does not guarantee both high conductivity and low crossover. The connectivity and chemistry of electrolyte‐equilibrated pathways, as well as defects at framework or polymer interfaces, must also be considered.

Non‐perfluorosulfonated rigid membranes represent a distinct separator class for RFBs, where shape‐persistent hydrocarbon backbones and intrinsic microporosity define sub‐nanometer transport pathways. When sulfonic‐acid sites are positioned within these rigid pores, ion conduction can remain continuous while crossover is reduced, motivating the rigid‐channel designs summarized in this section. Zuo et al. [65] employed sulfonated polymers with twisted, rigid backbones to restrict conformational freedom and promote dense packing, thereby generating confined ionic channels. By introducing hydrophobic, fluorinated moieties as swelling‐resistant domains with hydrophilic –SO_3_H groups, the membranes enabled rapid cation transport while selectively excluding redox‐active molecules, delivering excellent cycling stability in aqueous alkaline quinone RFBs (Figure 14a). In a complementary microporosity‐engineering strategy, Wong et al. [82] incorporated triptycene units into PEEK and subsequently carried out controlled sulfonation, the resulting intrinsically microporous ionomer increased free volume and formed highly interconnected water channels, supporting efficient dual‐ion transport of cations and OH^−^ (Figure 14b). By extending rigid‐channel design to imide frameworks, Chu et al. [156] synthesized microporous sulfonated polyimides with graded –SO_3_H distributions via single‐step polymerization. High molecular weight, moderate microporosity, and tuned interchain spacing yielded strong discrimination between protons and vanadium species, with the charged polyimide network acting simultaneously as proton pathways and vanadium barriers (Figure 14c). Precise sulfonation inside preformed micropores was demonstrated by Ye et al. [81], who used silyl‐protected sulfonating agents to functionalize a spirobifluorene‐based PIM (PIM‐SBF). This introduced cation‐conducting functionality while preserving the polymer's high rigidity and interconnected sub‐nanometer channels, thereby blocking redox‐active small molecules while permitting salt ion transport (Figure 14d). Branchi et al. [157] prepared polystyrene nanoparticles (via Friedel–Crafts alkylation and post‐functionalization) and embedded them into sulfonated poly(phenylene sulfone). The use of organic particles affords facile control over the sulfonation degree and hence IEC, enabling fine‐tuning of transport in solution‐casting membranes (Figure 14e). Wang et al. [158] fabricated PBI/COF hybrids in which crystalline TpPa and STpPa confer ordered sub‐nanometer pores that act as “proton highways.” Sulfonated STpPa further supplies additional hopping sites while serving as a vanadium‐blocking scaffold, collectively enhancing ion selectivity (Figure 14f). Across the studies summarized, non‐fluorinated sulfonated rigid membranes converge on a shared design logic: (i) fixing the channel geometry via twisted backbones, intrinsically microporous architectures (triptycene/PIMs), or crystalline fillers (such as COFs), thereby limiting swelling and preserving sub‐nanometer connectivity; (ii) locally programming ion chemistry by positioning –SO_3_H within or along these rigid pores to supply carrier density and hopping sites; (iii), decoupling charge‐carrier percolation from solute rejection such that percolated proton pathways are maintained while size/Donnan discrimination rejects multivalent ions and redox‐active molecules. When these elements are co‐optimized, the membranes simultaneously raise conductivity and sharpen selectivity, and they do so across both acidic (VRFBs) and alkaline (quinone) systems. For aqueous organic redox couples, selectivity should be assessed for the relevant molecular charge states and operating pH, since size exclusion alone may not predict the partitioning or adsorption of each species. In short, rigidity builds the channel, sulfonation governs the charge‐carrier transport, and their coupling dictates the selectivity–conductivity balance [159].

**FIGURE 14:**
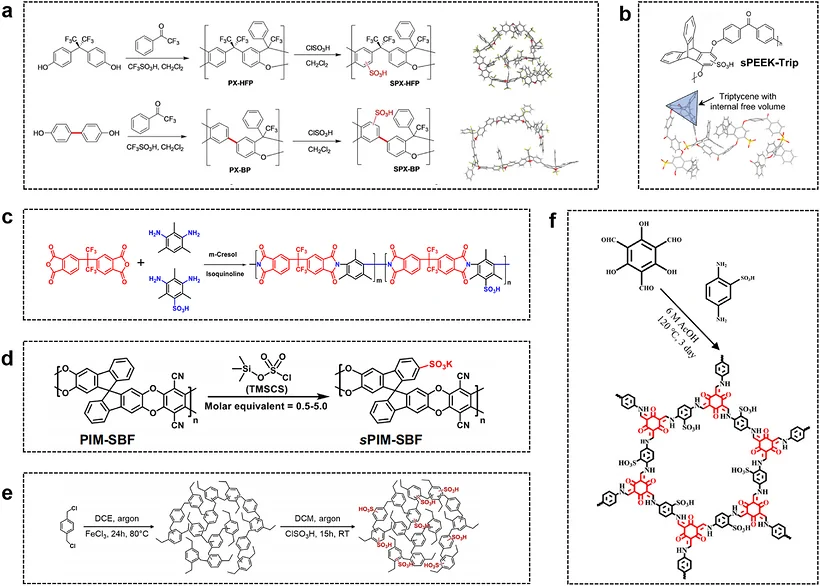
(a) Sulfonated polymer with rigid, twisted backbones to restrict polymer packing. Reproduced with permission [65]. Copyright 2020 John Wiley and Sons. (b) Designing microporous polymer via controlled sulfonation. Reproduced with permission [82]. Copyright 2025 Cell Press. (c) One‐step polymerization for polymer synthesis. Reproduced with permission [156]. Copyright 2023 Elsevier. (d) Precision‐controlled sulfonation using silyl‐protected sulfonating agents. Reproduced with permission [81]. Copyright 2024 Elsevier. (e) Synthesis of polystyrene nanoparticles via Friedel‐Crafts alkylation. Reproduced with permission [157]. Copyright 2018 Elsevier. (f) Incorporating crystalline COFs. Reproduced with permission [158]. Copyright 2014 Elsevier.

Translating these rigid‐channel designs to large‐area membranes presents different challenges for self‐supporting frameworks and filler‐containing polymers. Self‐supporting frameworks require continuous growth or assembly across the membrane area, control of grain boundaries and pinholes, and sufficient wet toughness for handling and stack sealing. Local structural order cannot establish film‐wide continuity. In polymer composites, filler sedimentation, dispersion stability, casting‐solution viscosity, and drying‐induced rearrangement may lead to spatial variations in filler loading, thickness, and transport. Supported thin selective layers and continuous coating offer possible processing routes, but introduce requirements for layer–support adhesion, low support resistance, and defect control. Small‐cell results should therefore be complemented by reporting membrane area, fabrication yield, spatial uniformity, batch reproducibility, and performance under representative stack compression and electrolyte flow conditions.

Non‐fluorinated sulfonated rigid membranes offer: (i) structural stability, where backbone rigidity and microporosity provide stable, ordered nanochannels with minimal dimensional change; (ii) chemical tunability, enabling precise placement of –SO_3_H to couple high IEC with continuous hopping networks, and the use of organic/COF fillers to tailor pore size and surface fields; (iii) selective transport, arising from sub‐nanometer steric control and Donnan exclusion that limit multivalent/organic crossover while sustaining cation conduction; and (iv) processing versatility and potential sustainability. Hydrocarbon matrices are readily modified and formulated and can be cast from solution, which can facilitate composite fabrication, although scale‐up and cost depend on filler dispersion, framework precursor prices, synthesis and purification requirements, and fabrication yield. These attributes together provide a coherent pathway to decouple conductivity from crossover, while retaining mechanical integrity and compatibility with practical cell operation. Lower crossover can result from reduced uptake of the active species, slower motion within the membrane, or both. Permeability data alone cannot separate these contributions; complementary sorption, transport, and hydrated‐structure measurements are needed. In composite membranes, filler porosity and surface functionality influence transport through their interaction with the polymer matrix. Hydrogen bonding, electrostatic or acid–base interactions, and, where present, covalent attachment may improve filler dispersion, modify local hydration, or constrain polymer rearrangement. Their effects are conditional: strong ion association may hinder carrier mobility, whereas poor compatibility or aggregation can create interfacial voids and nonselective pathways. The stability of a COF or MOF also depends on its specific linkages or metal–ligand coordination and the operating electrolyte; it should not be inferred from the framework label alone.

## 6 Summary and Outlook

Sulfonic acid‐based polymer networks remain the foundational platform for RFB membranes, but realizing application‐level performance hinges on strategies that simultaneously deliver high conductivity and strong selectivity. Recent mechanistic insights indicate that precisely programmed intrinsic microporosity, together with controlled grafting and crosslinking, can establish percolated proton pathways while restricting the crossover of redox‐active species. In parallel, fluorine‐free aromatic matrices, particularly when hybridized with inorganic reinforcements, offer an attractive balance of chemical robustness and cost advantages. Nonetheless, swelling‐induced performance loss and long‐term degradation under electrochemical and thermal stresses persist as central bottlenecks. Long‐term durability should be assessed by distinguishing chemical changes in the polymer backbone and ionic groups from mechanical damage, filler loss, and interfacial debonding. Repeated swelling may couple these failure modes. Aging studies should therefore track chemical and morphological changes alongside membrane resistance, active‐species crossover, and wet mechanical properties.

The studies discussed above suggest six practical design rules. First, define the intended charge carrier and the redox‐active species to be retained before selecting membrane charge and pore chemistry. Second, control the spatial distribution and accessibility of –SO_3_H rather than maximizing its content alone. Third, maintain connected ionic pathways while limiting excessive water uptake and swelling. Fourth, combine structural reinforcement with adequate compatibility at polymer–filler interfaces. Fifth, optimize membrane thickness together with the selective layer and its support, since both affect resistance and crossover. Sixth, evaluate these choices under the intended electrolyte and operating conditions, while considering reproducibility and manufacturing feasibility. These rules require application‐specific trade‐offs. Higher sulfonation may improve hydration and charge‐carrier transport, but excessive swelling can increase active‐species crossover. Crosslinking and rigid scaffolds may stabilize hydrated domains, whereas excessive constraint can hinder ion mobility, toughness, or film processing. Thinner membranes reduce area‐specific resistance but are more sensitive to pinholes and may permit greater crossover. Combining controlled sulfonation with moderate reinforcement may help preserve connected pathways while limiting swelling, provided that the interface remains stable. Likewise, a thin selective layer on a robust support may reduce resistance without sacrificing handling strength, provided that layer defects, adhesion, and support resistance are controlled.

Future progress will depend on diversifying sulfonic acid–based chemistries via hybridization and coupling with advanced design tools, including surface/interfacial modification and the construction of multifunctional layered architectures [160]. Biomimetic nanochannel architectures that emulate biological ion selectivity hold promise for simultaneously achieving high ionic conductivity and ultralow ion permeability. For example, natural aquaporins provide prototypical sub‐nanometer water channels that combine ultrafast water transport with strong ion exclusion, offering a concrete blueprint for constructing highly confined, hydration‐structured pathways within sulfonated matrices. On the synthetic side, bio‐inspired concepts have already been realized in systems such as artificial water‐channel polymers and sub‐nanoporous graphene membranes, where angstrom‐scale pores decorated with specific functional groups afford strong ion selectivity at comparatively low area‐specific resistance. These proof‐of‐concept nanochannel membranes suggest that similar confinement and chemical‐patterning strategies could be integrated into sulfonic‐acid‐based matrices to suppress crossover of bulky redox‐active species while retaining efficient ion transport. Machine learning and high‐throughput screening could help identify promising formulations if datasets include descriptors for polymer structure, –SO_3_H content and distribution, filler chemistry, processing conditions, and electrolyte composition. Optimization should consider resistance, crossover, swelling, durability, and cost together. Given the limited size and inconsistent testing conditions of existing datasets, predicted improvements require validation under comparable experimental conditions. At the same time, stability must be strengthened through covalent tethering, interchain reinforcement, and scaffolded frameworks capable of withstanding concentrated electrolytes and temperature fluctuations [161]. For example, by introducing side‐chain –SO_3_H groups anchored through robust C─C bonds, embedding sulfonated blocks into mechanically reinforced backbones, or constructing inorganic–organic hybrid frameworks where sulfonated polymer domains are confined within silica, MOFs or oxide networks that suppress swelling and enhance oxidative and thermal durability.

Translating materials advances into practice requires rigorous, comparable testing and manufacturability. Scale‐up requires control over batch‐to‐batch variations in sulfonation and polymer molecular weight, casting‐solution stability, drying conditions, film thickness, and defect formation. Cost assessments should include functionalization, solvent recovery, drying, quality control, and manufacturing yield rather than raw‐material price alone. Standardized protocols should quantify calendar and cycle life under realistic electrolytes and operating profiles, while techno‐economic metrics assess roll‐to‐roll scalability and cost‐effectiveness, thereby enabling a more objective evaluation of practical applicability. For scale‐up claims, evaluation should also report tested membrane area, thickness, and defect uniformity, batch‐to‐batch yield, and performance under representative stack compression and electrolyte flow conditions. Meaningful comparisons should report membrane pretreatment, hydrated thickness, electrolyte composition, temperature, and the species and direction of transport measured [162]. Cell tests should also specify current density and other relevant operating conditions, report elapsed time alongside cycle number, and examine whether capacity loss arises from crossover, electrolyte degradation, or both [163]. Integrating advanced characterization with multiscale modelling will accelerate the establishment of quantitative structure‐transport–lifecycle relationships tailored to specific RFB chemistries. Key unresolved questions are how ions partition and move within membranes exposed to concentrated electrolytes, how hydrated transport pathways evolve during operation, and whether selective transport can be retained across large‐area films with reproducible thickness and few defects. By aligning molecular design, process scalability, and durability assessment, sulfonic acid–based membranes are well‐positioned to enable high‐power‐density RFBs for grid‐scale renewable integration and long‐duration storage—an essential step toward deep decarbonization (Figure 15).

**FIGURE 15:**
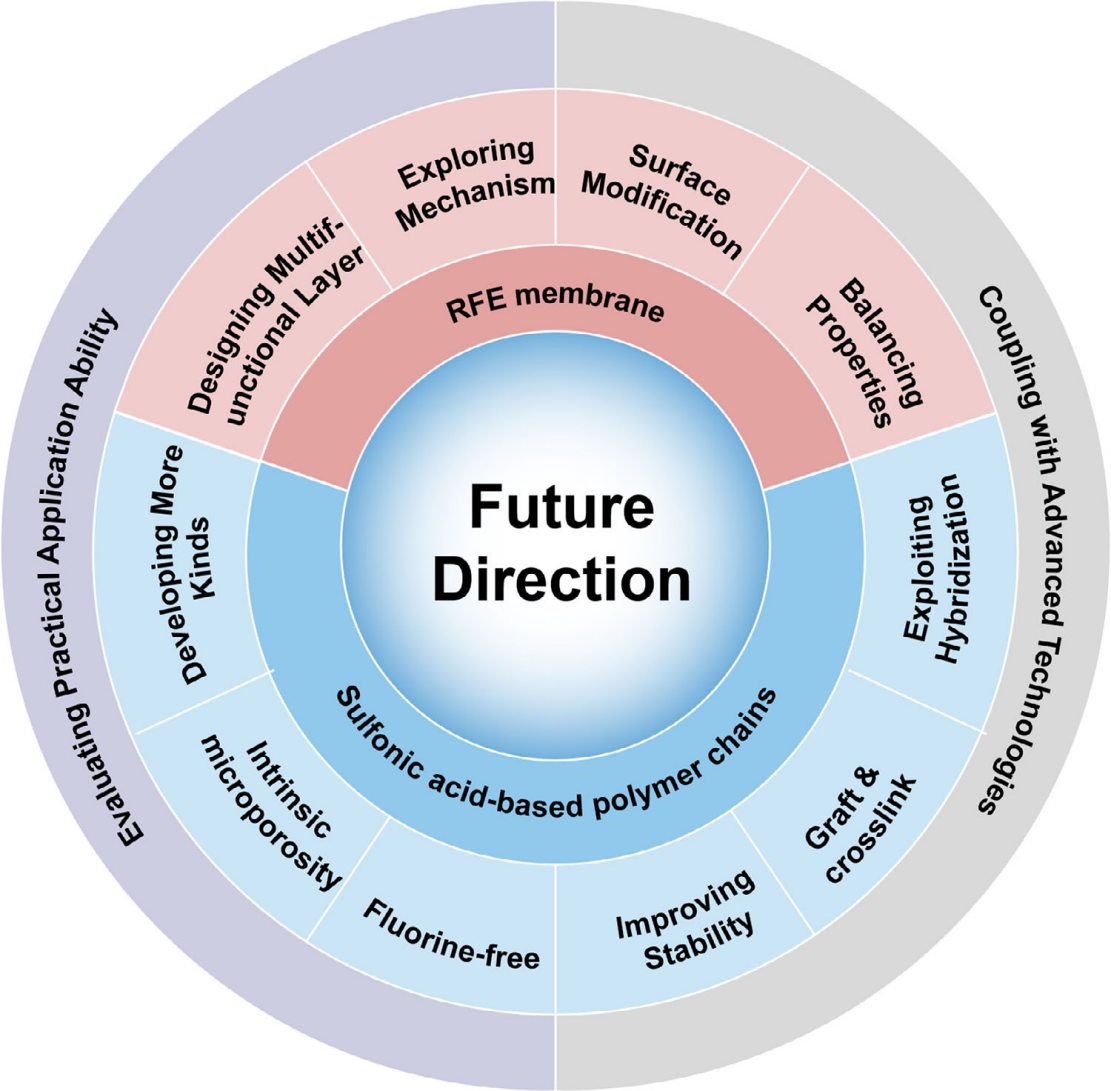
Schematic of future direction of sulfonated polymer in RFBs.

## Author Contributions

**Jinkang Liu**: writing – original draft, methodology, investigation, formal analysis, data curation. **Mingbo Xie**: writing – original draft, methodology, investigation, formal analysis, data curation. **Zhenghao Wang**: writing – original draft, formal analysis. **Huacun Li**: methodology, investigation. **Jun Xie**: writing – review and editing. **Pan Liu**: writing – review and editing. **Qingwei Wang**: writing – review and editing. **Xu Yan**: writing – review and editing. **Meiqing Shi**: writing – review and editing, project administration. **Liyuan Chai**: funding acquisition, writing – review and editing, supervision. **Yue Luo**: writing – review and editing, formal analysis. **Zining Tang**: writing – review and editing, methodology. **Rui Tan**: conceptualization, writing – review and editing, supervision, funding acquisition, formal analysis. **Liyuan Zhang**: writing – review and editing, supervision, formal analysis, funding acquisition.

## Funding

This work is supported by the National Key R&D Program of China (2024YFC3907602), Jiangxi Province Introduced and Co‐built R&D Institutions (20244BCF61002), Key R&D Program of Jiangxi Province, China (20252BCF320029), the science and technology innovation Program of Hunan Province (2025RC3034), and R. T. acknowledges the support from the Royal Society (RGS∖R2∖252134).

## Conflicts of Interest

The authors declare no conflicts of interest.

## Data Availability

All the data shown in Figures 8, 9, 10, 11, 12, 13, 14 are obtained from previous publications with retrieved copyrights. No original data are used or presented in this review article.
